# Cold Shock for Cold Tolerance: Phytohormone Dynamics in Sorghum Provides Insights

**DOI:** 10.1002/pld3.70133

**Published:** 2026-01-15

**Authors:** Luisa Neitzert, Natalja Kravcov, Yudelsy Antonia Tandron Moya, Steffen Windpassinger, Nicolaus von Wirén, Rod Snowdon, Benjamin Wittkop

**Affiliations:** ^1^ Department of Plant Breeding, IFZ Research Center for BioSystems, Land use and Nutrition Justus‐Liebig University Giessen Giessen Germany; ^2^ Department Molecular Plant Nutrition Leibniz Institute of Plant Genetics and Crop Plant Research (IPK) OT Gatersleben Germany

**Keywords:** abscisic acid, climate adaptation, cold sensitivity, gibberellins, jasmonic acid, pollen fertility, reproductive cold tolerance, *sorghum bicolor*

## Abstract

The loss of yield due to cold stress during the early reproductive phase poses challenges to the expansion of sorghum cultivation into temperate regions. A better understanding of the physiological mechanisms is crucial for rapid progress in breeding cold‐tolerant sorghum varieties. To identify the floral phytohormones responsible for reproductive cold tolerance, a cold‐tolerant and a cold‐sensitive genotype were subjected to cold stress at various developmental stages during the early reproductive phase. In addition to abscisic acid and its derivatives, including abscisic acid glucose ester, dihydrophaseic acid, and phaseic acid, various gibberellins as well as jasmonic acid and its bioactive form jasmonic acid isoleucine were examined. We found that cold‐tolerant sorghum is capable of downregulating abscisic acid concentration under cold stress. While existing literature primarily attributes increased abscisic acid concentration, combined with an insufficient pool of bioactive gibberellins, in sensitive plants as a result of abnormal pollen development, this study shows that this is not the case in sorghum. Additionally, an antagonistic interaction between gibberellins and jasmonic acid was observed regardless of genotype and environmental conditions. These findings contribute to a better understanding of the physiological mechanisms behind cold tolerance in sorghum and could provide important insights for future breeding efforts aiming to accelerate the expansion of cold‐tolerant sorghum varieties into temperate climates.

## 1 Background

Plants are able to adapt to stressful conditions through changes in physiological processes (Bohnert et al. 1995). A particular focus is on the adaptation of sorghum to cold, which is essential for its expansion into temperate climates. In times of climate change, increasing population pressure, and deteriorating soil quality, sorghum is gaining importance as a robust crop to ensure food and feed supply (Bindraban et al. 2012; Patil 2017). Sorghum presents a promising option for agriculture in temperate regions due to its high adaptability, including the ability to thrive under dry conditions, efficient water usage, and growth on less fertile soils (Berenji and Dahlberg 2004; Zheng et al. 2011; Patil 2017). The added value provided by sorghum cultivation is increasingly sparking interest from both scientists and agronomists to decipher the mechanisms contributing to cold tolerance to expand cultivation in the future. While juvenile cold tolerance is well researched, there is limited knowledge at the physiological level regarding cold tolerance during the reproductive stage. There is a significant need for further research regarding the physiological mechanisms contributing to cold tolerance, particularly phytohormone regulation in sorghum. Abscisic acid (ABA) and gibberellins (GA), both key hormones in cold stress, have already been discovered in rice plants. While cold‐tolerant rice plants could maintain low ABA levels through the presence of ABA‐regulating genetic sequences (*OSZEP1* and *OSNCED3*), the ABA concentration in cold‐sensitive plants was significantly higher (Oliver et al. 2007; Ji et al. 2011; Sharma and Nayyar 2016). Furthermore, increased degradation of ABA through conjugation or oxidation in cold‐tolerant rice plants is thought to reduce ABA levels. Here, the expression of both ABA‐8‐hydroxylase genes, *ABA8ox1* and *ABA8ox2*, is higher in cold‐tolerant genotypes (Nambara and Marion‐Poll 2005; Oliver et al. 2007). In addition to ABA regulation, maintaining a sufficiently high pool of bioactive gibberellins to protect the plant from cold damage has been identified, with altered biosynthesis pathways of GA4 and GA7 (Sharma and Nayyar 2016). Besides altered ABA and GA regulation, jasmonic acid is also attributed a key role in involvement in phytohormones contributing to cold tolerance in different poaceae species (Du et al. 2013; Cai et al. 2014). However, problems with hypertrophy of tapetal cells and abnormal pollen growth are described as a result of increased ABA levels and insufficient pools of bioactive gibberellins in the absence of cold tolerance. Suppression of the cell wall‐bound acid invertase gene and the monosaccharide transporter gene disrupts sugar transport to the tapetum, resulting in abnormal pollen development and reduced grain number (Oliver et al. 2005; Mamun et al. 2006). In contrast, reduced fertility of the female floral organ has been discussed as a possible cause for the reduced grain set in cold‐sensitive genotypes (Osuna‐Ortega et al. 2003, Neitzert et al. 2025).

This work aims to gain a deeper understanding of the physiological mechanisms related to phytohormones in reproductive cold tolerance in sorghum by addressing several open questions: (I) Which phytohormones are down‐ or up‐regulated under cold stress? (II) What differences exist in phytohormone response between cold‐tolerant and cold‐sensitive genotypes? (III) How do phytohormone levels differ between developmental stages during the early reproductive phase? (IV) To what extent can these results be transferred from existing literature on related species? Overall, these findings will contribute to a better understanding of the physiological mechanisms behind cold tolerance in sorghum and could provide important insights for future breeding of cold‐tolerant sorghum varieties to accelerate their expansion into temperate climates.

## 2 Material and Methods

### 2.1 Plant Material

**FIGURE 1:**
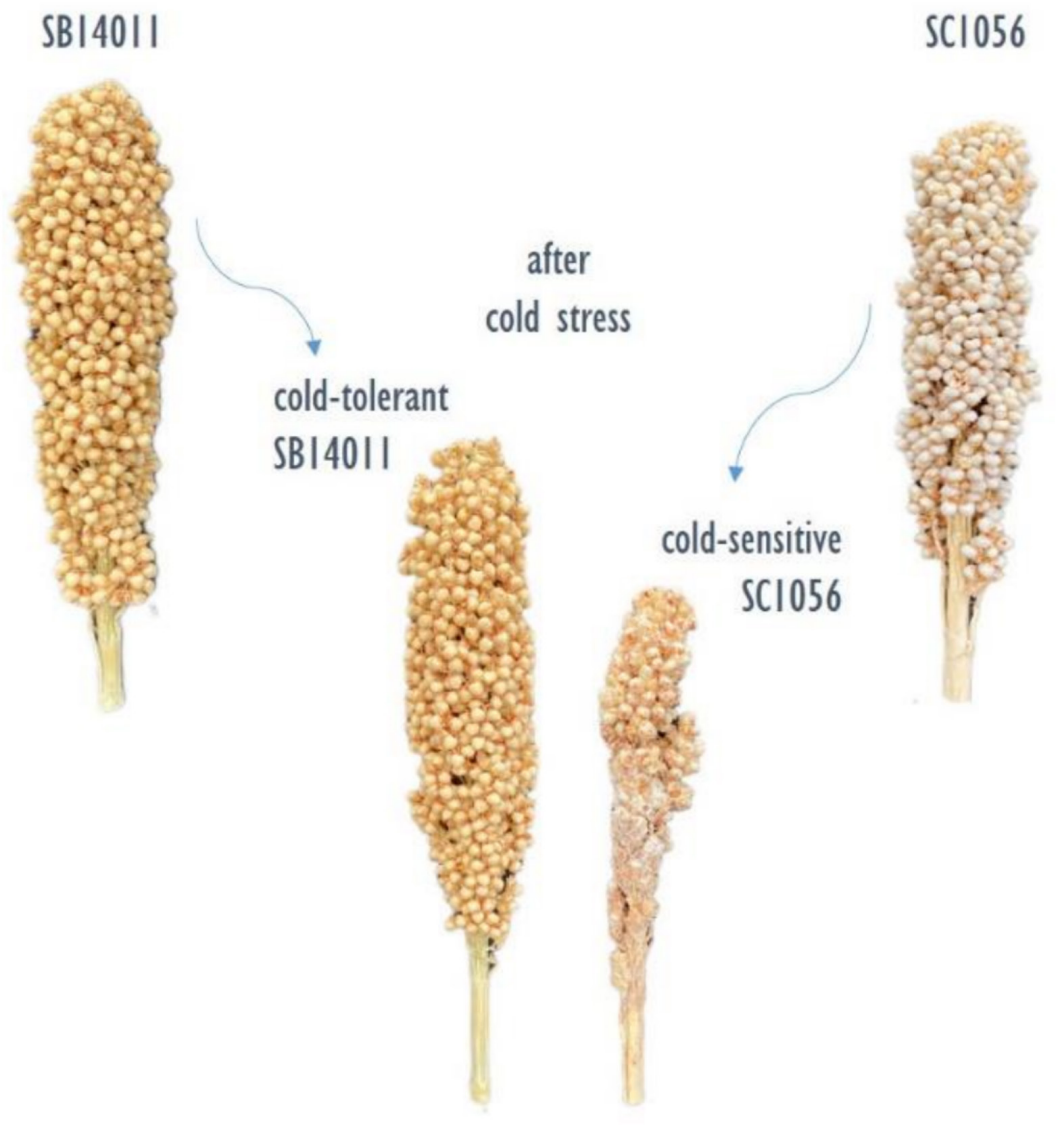
cold‐tolerant parental line SB14011 and cold‐sensitive parental line SC1056 grown under optimal and stress conditions (see Table 1).

For the experiments, one cold‐tolerant (SB14011) and one cold‐sensitive (SC1056) inbred line were studied (see Figure 1). The two inbred lines were selected from a diversity set of *Sorghum bicolor* (*n* = 330) consisting of inbred lines of different origin, utilization and subspecies. Traits such as seed yield and panicle harvest index (PHI) under cold conditions were used to evaluate reproductive cold tolerance (Chakrabarty et al. 2021). Thus, genotype SB14011, a breeding line developed and selected under german conditions, was identified as cold‐tolerant and SC1056, a conversion line originating from Sudan (Stephens et al. 1967), as cold‐sensitive.

**TABLE 1:** Settings of the climate chamber experiment.

	Temperature in Celsius	Day/night cycle in hours	Rel. air humidity
Optimum conditions	30/24	13/11	60%
Cold stress	25/7	13/11	60%

### 2.2 Defining BBCH‐Stages

**FIGURE 2:**
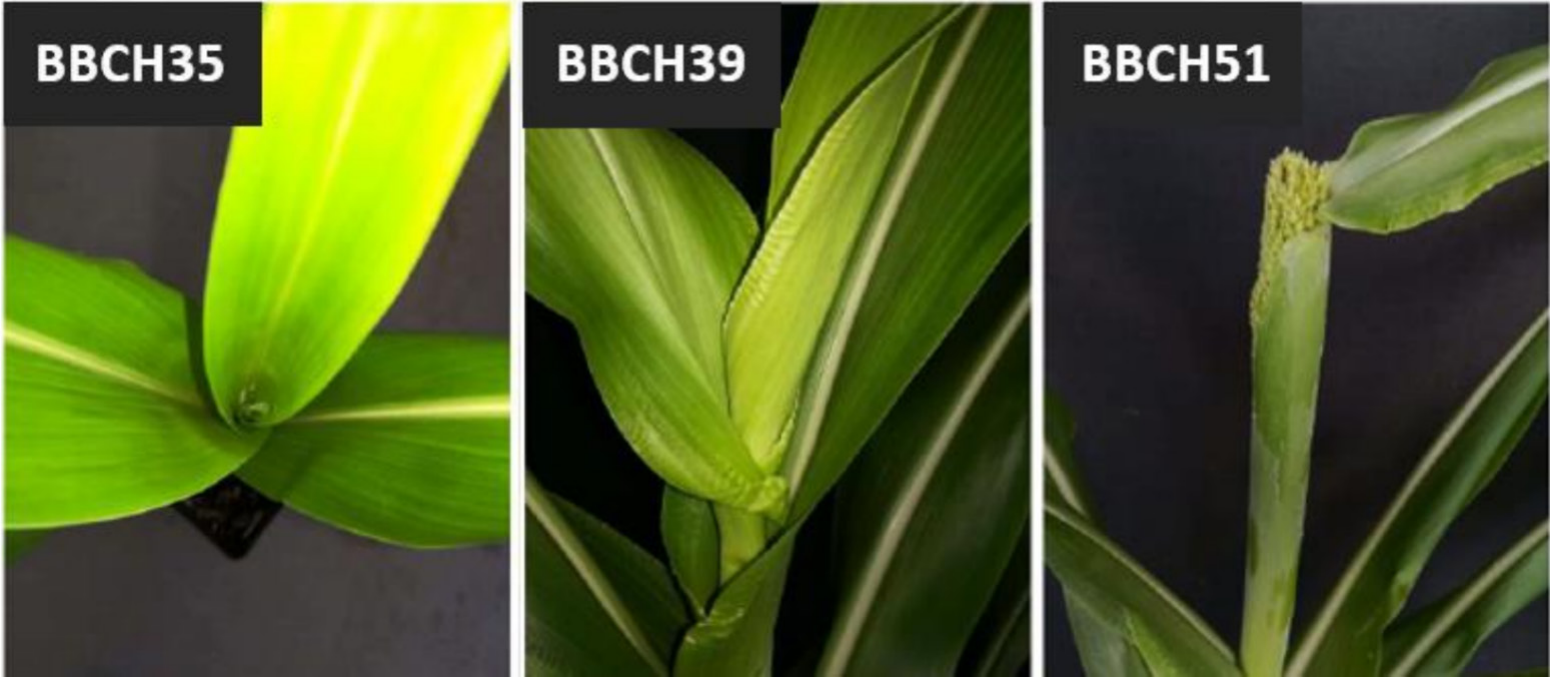
Overview of the developmental stages to be examined—BBCH35: flag leaf not yet visible, shortly before the appearance of the leaf tip; BBCH39: ligule of the flag leaf visible; BBCH51: emergence of the panicle.

In this study, we investigated the effects of cold exposure on plants at specific developmental stages. The BBCH scale was used for the standardized coding of these stages. This scale is based on the cereal scale developed by Zadoks et al. (1974) and subdivides plant development into macro‐ and microstages. The currently applied extended BBCH scale was jointly developed by the Federal Biological Research Centre for Agriculture and Forestry (BBA), the Federal Plant Variety Office (BSA), and the Agricultural Industry Association (IVA). The acronym BBCH is derived from these german institutions: **B** iologische Bundesanstalt für Land‐ und Forstwirtschaft, **B** undessortenamt, and **Ch** emische Industrie. In this study, the effects of cold were analyzed starting from BBCH35, BBCH39, and BBCH51 (see Figure 2). At BBCH35, the plants had reached the 5‐node stage, indicating that the 5th node was at least 2 cm away from the 4th node. During this stage, the flag leaf was notably small, positioned relatively low, and tightly curled up. At BBCH39, the flag leaf had fully developed, exhibiting visible ligules. Finally, at BBCH51, the heading process had initiated, characterized by the emergence of the panicle tip from the leaf sheath (according to the Technology and Promotion Centre (TFZ), BBCH code for sorghum).

### 2.3 Climate Chamber Experiments

The climate chamber experiments were conducted at the IFZ Research Center for Biosystems at Justus Liebig University in Giessen, Germany following the procedure described by Schaffasz et al. (2019) with minor modifications. The genotypes were tested under controlled cold stress and optimal growth conditions at different developmental stages (BBCH35, BBCH39, and BBCH51) in the reproductive stage. The plants were grown in 17 × 17 × 17 cm pots filled with Fruhstorfer type N soil and were irrigated and fertilized following good horticultural practices to exclude variation in conditions other than thermal stress. All plants were cultivated under optimal conditions (30°C/13 h during the day and 24°C/11 h at night, with a relative humidity of 60% and LED lighting) during emergence and early vegetative growth. For each of the mentioned BBCH stages, randomly selected plants were transferred to a cold stress chamber (25°C/13 h during the day and 7°C/11 h at night, with a relative humidity of 60% and LED lighting) and exposed to cold stress for 3 days. Three plants were stressed for each genotype and BBCH stage. Under optimal conditions, the panicles were harvested 1 day after reaching the respective BBCH stage. The individual panicles were considered biological replicates, with two technical replicates each for statistical analysis.

### 2.4 Phytohormone Analysis

The phytohormones analyzed were abscisic acid (ABA), abscisic acid glucose ester (ABAGe), dihydrophaseic acid (DHPA), phaseic acid (PA), jasmonic acid (JA), jasmonic acid isoleucine (JAIle), gibberellins (GA). Gibberellins GA1, 3, 4, 5, 6, 7, 8, 9, 20, 29, 51 were included in the analysis but were not detected in the samples. GA 12, 15, 19, 24, 44, 53 were detected. Organic solvents, methanol (MeOH) and acetonitrile (ACN), were purchased from Th. Geyer GmbH & Co. KG, Germany. Formic acid (FA) was obtained from Biosolve Chimie, France. De‐ionized distilled water from the Milli‐Q Reference System, Merck, Germany, was used for preparation of all mixtures and as mobile phase.

Standards ABA, ABAGe, JA, JAIle, and GA as well as deuterium labeled compounds used as internal standards were purchased from OlChemim s.r.o, Czech Republic. Standards DHPA and PA were purchased from the National Research Council of Canada, Saskatoon, Canada.

Plant material was ground in liquid nitrogen. Samples of ca. 30 mg (fresh weight) were weighed into 2 mL safe lock tubes (Eppendorf AG, Germany) and kept at −80°C until extraction. Empty tubes were used as blanks. Before extraction, two 3 mm ceria‐stabilized zirconium oxide beads were placed into each tube. The samples were extracted and purified as described in Šimura et al. (2018) with some modifications. Briefly, 1 mL ice‐cold 50% aqueous (v/v) ACN containing the internal standards was added to each tube for phytohormone extraction. Samples were homogenized in a MM 301 vibration mill (Retsch GmbH, Germany) operating at a frequency of 27 Hz for 5 min and afterward sonicated for 3 min at 4°C using a Sonorex ultrasonic bath (BANDELIN electronic GmbH, Germany). Samples were subsequently extracted using a Reax 32 overhead shaker (Heidolph Instruments GmbH, Germany) for at least 30 min. After 10 min centrifugation at 14000 rpm and 4°C (CT 15 RE centrifuge, Himac, Japan), the supernatant was transferred to clean Eppendorf tubes. All samples were purified using Oasis PRIME HLB RP (1 cc per 30 mg), polymer‐based SPE cartridges (Waters Co., USA). After loading the supernatant, the flow‐through fraction was collected in a clean tube. The cartridge was then rinsed with 1 mL of 30% (v/v) ACN, and this fraction was collected in the same tube as the flow‐through fraction. After this single‐step SPE, the samples were evaporated to dryness at 40°C in a vacuum concentrator RVC 2–33 IR (Martin Christ GmbH, Germany) and stored at −20°C until analysis. For analysis, the samples were dissolved in 50 μL of 30% ACN (v/v) containing 0.1% FA and transferred to insert‐equipped vials. The analysis of targeted phytohormones was performed by Ultra High Pressure Liquid Chromatography‐Heated Electrospray Ionization‐High Resolution Mass Spectrometry (UHPLC‐HESI‐HRMS) technique.

Compound baseline separation was achieved on a reversed phase Acquity UPLC HSS T3 column (10 Å, 2.1 × 100 mm, 1.8 μm, Waters) using a simple gradient elution of A (Water, 0.1% FA) and B (ACN, 0.1% FA) as follows: 0–5 min, 10% B; 5–10 min, 10% to 80% B. Five additional minutes were added for column washing and re‐equilibration. In the case of GA, the gradient elution consisted of A (Water, 0.1% FA) and B (MeOH, 0.1% FA) as follows: 0–0.3 min, 10% B; 0.3–0.7 min, 10% to 30% B; 0,7–2 min, 30% to 50% B; 2–4 min, 50% to 60% B; 4–8 min, 60% to 80% B; 8–9.5 min, 80% to 99% B; 9.5–10,4 min, 99% B. The column temperature was set at 45°C and the flow rate at 0.5 mL/min (0.3 mL/min for GA). To preserve the integrity of the column a guard column (130 Å, 2.1 × 5 mm, 1.8 μm, Waters) was used. The injection volume was 5 μL. The UHPLC system (Vanquish Horizon, Thermofisher, San Jose, CA, USA) was coupled to a Q Exactive Plus Mass Spectrometer (Thermofisher, San Jose, CA, USA) equipped with a HESI source operating in negative ion mode. Source values were set as follows: Spray voltage 2.5 kV; Capillary temperature 255°C; S‐lens RF level 40; Aux gas heater temp 320°C; Sheath gas flow rate 47; Aux gas flow rate 11. For spectra acquisition a FullMS/dd‐MS^2^ experiment was performed. Resolution in Full Scan was set as 70,000. For MS/MS experiments resolution 17,500 and NCE 40 V were used. MS data were acquired and processed by Trace Finder Software (v. 4.1, Thermo Scientific, San Jose, CA, USA). Twelve‐point curve was prepared from standards mix solutions in the range of 0.5 to 1000 nM. To generate the calibration curve, the peak area on the extracted ion chromatogram (XIC) of the deprotonated molecule ion [M‐H]‐ was measured. A least‐square linear regression was used to best fit the linearity curve.

### 2.5 Statistical Analysis

A Welch two‐sample *t*‐test was conducted to analyze significant differences in the investigated phytohormone concentrations between stress treatment and optimal conditions at specific treatment stages (BBCH35, BBCH39, BBCH51) separately for each genotype.

To test for differences between the two genotypes, a paired *t*‐test was performed to compare each individual treatment stage (BBCH35, BBCH39, and BBCH51). This was done separately for the two conditions (cold stress and optimal conditions).

Separately, a one‐way ANOVA analysis was conducted for each trait to assess possible differences between treatment stages (BBCH35, BBCH39, BBCH51) within each genotype. This was also done separately for the two conditions (cold stress and optimal conditions). The independent variable in each analysis was “treatment”, and the dependent variable was the respective trait. These traits included the phytohormones abscisic acid, abscisic acid glucose ester, dihydrophaseic acid, phaseic acid, jasmonic acid, jasmonic acid isoleucine, gibberellins (gibberellic acid 12, 15, 19, 24, 44, and 53).

After each ANOVA, a Least Significant Difference (LSD) post hoc analysis was performed to determine which treatments showed statistically significant differences from each other.

The Pearson correlation coefficients were used to evaluate the relationships between phytohormones, pollen traits, and yield parameters. Separate correlations were performed for the two genotypes (SB14011 and SC1056) and the two treatments (control and stress).

The statistical analysis and presentation of results were conducted in R version 4.0.5.

## 3 Results

### 3.1 Impact of Cold Stress on Phytohormone Levels

Significant differences in various phytohormone concentrations were seen at all developmental stages for both the cold‐tolerant genotype SB14011 and the cold‐sensitive genotype SC1056 during the control and stress treatments (Table 2).

**TABLE 2:** Results of the Welch two‐sample *t*‐test analyzing significant differences between control and stress conditions across various treatments (BBCH35, BBCH39, and BBCH51), separately for the genotypes SB14011 and SC1056, concerning different phytohormones, including abscisic acid (ABA), abscisic acid glucose ester (ABAGe), dihydrophaseic acid (DHPA), jasmonic acid (JA), phaseic acid (PA), gibberellins (GA), and its derivatives GA12, GA15, GA19, GA24, GA44, and GA53; (Significance level: *** 0.001; ** 0.01.; * 0.05).

Genotype	SB14011			SC1056		
Contrasts	BBCH35	BBCH39	BBCH51	BBCH35	BBCH39	BBCH51
Phytohormone	*p*					
ABA	0.1321	0.5938	0.067	0.0105*	8.57E‐05***	0.0064**
ABAGe	0.0574	0.1182	0.3632	0.2979	NA	0.009**
DHPA	0.6897	0.6602	0.0072**	0.3439	0.0584	0.8204
PA	0.0127*	0.0707	0.005**	0.0198*	0.0026**	0.8534
GA	0.8298	0.0022**	0.1826	0.16	0.0003***	0.02033*
JA	0.4113	0.6635	0.1615	0.2911	0.1098	0.0725
JAIle	0.42	0.1368	0.8257	0.2721	0.235	0.1645
GA12	0.1323	NA	NA	0.1531	NA	NA
GA15	0.0685	NA	NA	NA	NA	NA
GA19	0.5613	0.0016**	0.7392	0.101	9.02E‐05***	0.6145
GA24	0.0315*	NA	NA	0.042*	NA	0.3632
GA44	0.9153	0.0005***	0.2637	0.2264	0.0009***	0.1106
GA53	0.498	0.0349*	0.0346*	0.1412	0.0034**	0.8208

For genotype SB14011, at BBCH35 under stress conditions compared to the control, significant differences were observed exclusively in the concentration of phaseic acid (PA). In BBCH39, significant differences were observed in the concentrations of the gibberellin (GA) derivatives GA19, GA44, and GA53 depending on the conditions. Similarly, significant differences in PA concentration were observed at BBCH51, along with significant disparities in the concentrations of dihydrophaseic acid (DHPA) and GA53.

In the case of genotype SC1056, significant differences between control and stress conditions were observed for the hormones abscisic acid (ABA), PA, and the gibberellin derivatives GA24 at BBCH35. At BBCH39, significant differences in ABA, PA, GA, and the gibberellin derivatives GA19, GA44, and GA53 were observed depending on the treatment. At BBCH51, a significant difference in ABA concentration between stress and control conditions was also noted, along with significant differences in the concentrations of abscisic acid glucose ester (ABAGe) and GA.

**FIGURE 3:**
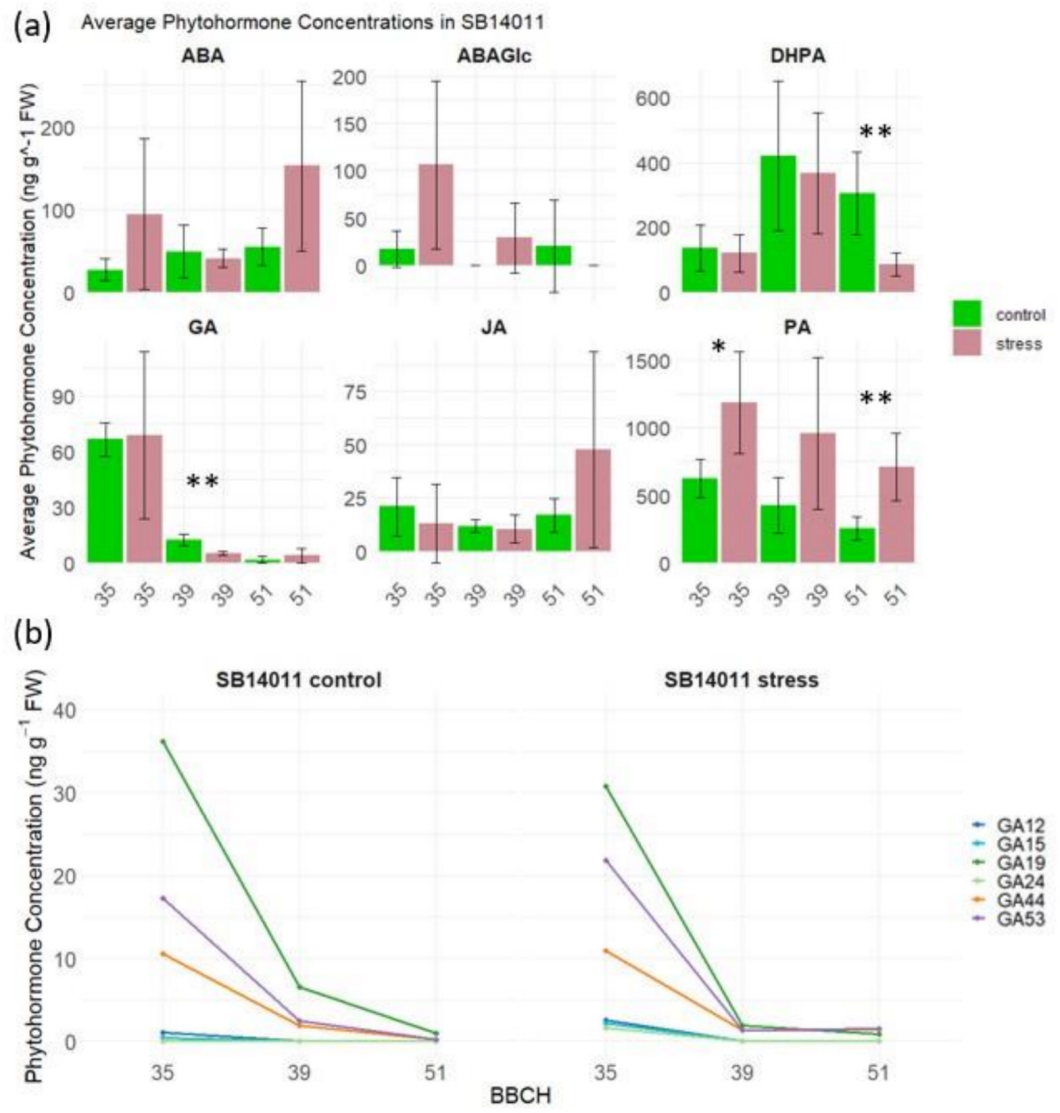
(a) Comparison of phytohormone concentrations including Abscisic Acid (ABA), Abscisic Acid Glucose Ester (ABAGe), Dihydrophaseic Acid (DHPA), Gibberellins (GA), Jasmonic acid (JA), and Phaseic Acid (PA) between stress and control conditions of genotype SB14011 at different developmental stages (BBCH35, BBCH39, and BBCH51); (b) Comparison of phytohormone concentrations of Gibberellin derivatives including GA12, GA15, GA19, GA24, GA44, and GA53 between stress and control conditions of genotype SB14011 at different developmental stages (BBCH35, BBCH39, and BBCH51).

The differences in phytohormone concentrations for the cold‐tolerant genotype SB14011 under stress and control conditions at various developmental stages (BBCH35, BBCH39, and BBCH51) are illustrated in Figure 3. The bar graphs highlight significant differences in phytohormone concentrations, including dihydrophaseic acid (DHPA), phaseic acid (PA), gibberellins (GA), and their derivatives between the two conditions.

At BBCH35, SB14011 exhibits a significantly higher PA concentration under stress conditions compared to the control. Conversely, at BBCH39, the GA concentration and the concentrations of its derivatives GA19, GA44, and GA53 are significantly lower under stress conditions. Additionally, at BBCH51, alongside the elevated PA and GA53 concentrations, a reduced DHPA concentration is observed compared to the control.

**FIGURE 4:**
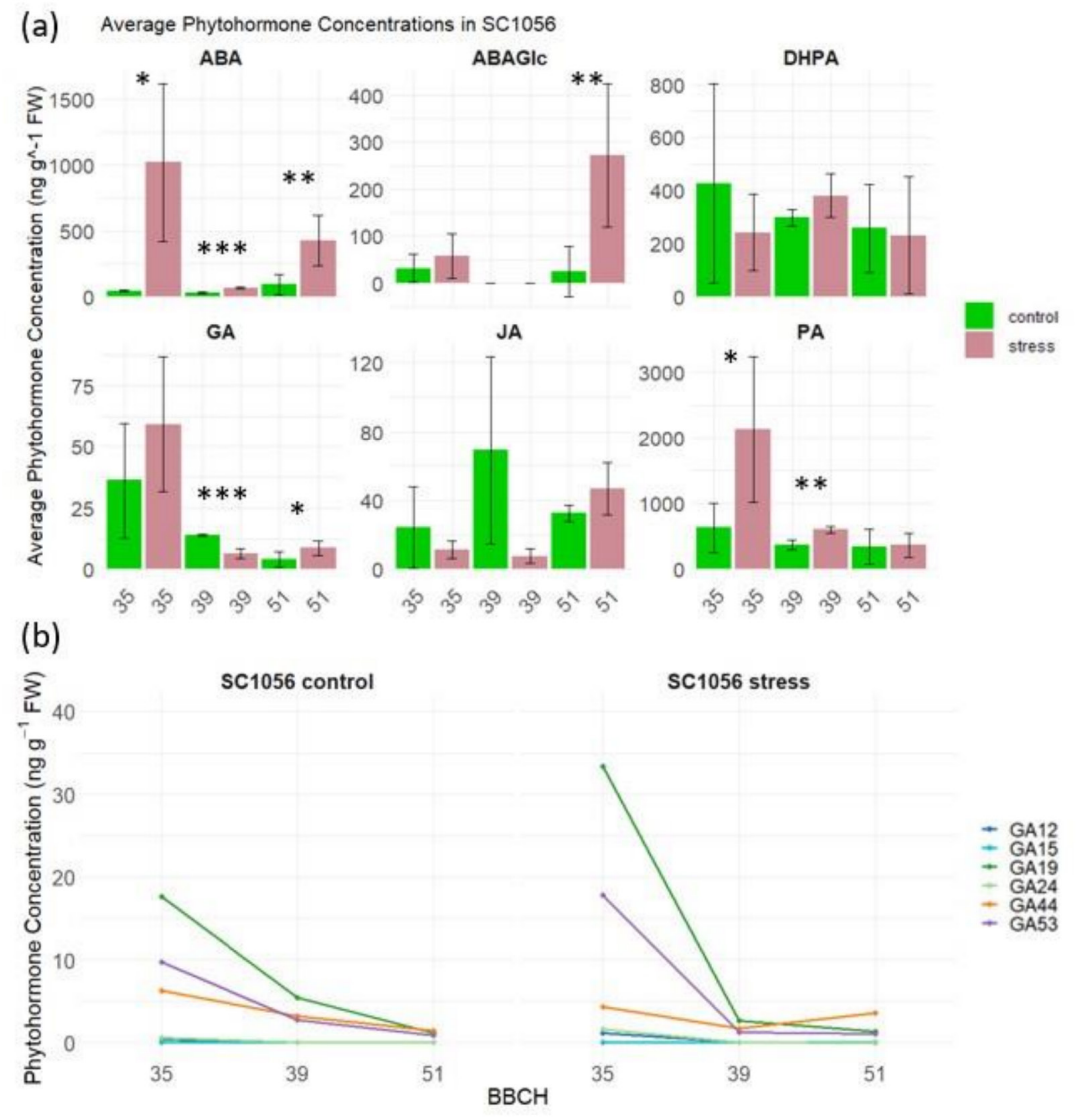
(a) Comparison of phytohormone concentrations including abscisic acid (ABA), abscisic acid glucose ester (ABAGe), dihydrophaseic acid (DHPA), gibberellins (GA), jasmonic acid (JA), and phaseic acid (PA) between stress and control conditions of genotype SC1056 at different developmental stages (BBCH35, BBCH39, and BBCH51). (b) Comparison of phytohormone concentrations of Gibberellin derivatives including GA12, GA15, GA19, GA24, GA44, and GA53 between stress and control conditions of genotype SC1056 at different developmental stages (BBCH35, BBCH39, and BBCH51).

Significant differences in the concentrations of abscisic acid (ABA), abscisic acid glucose ester (ABAGe), phaseic acid (PA), gibberelins (GA), and their derivatives are apparent when considering the phytohormone concentrations of the cold‐sensitive genotype in Figure 4 under stress versus control conditions across various developmental stages (BBCH35, BBCH39, and BBCH51). Particularly, the significantly higher ABA concentrations under stress conditions observed across all developmental stages are notable. Furthermore, at BBCH35, PA and GA24 concentrations are also significantly elevated under stress conditions. In addition to the increased ABA and PA concentrations, BBCH39 exhibits significantly lower GA concentrations under stress conditions. Furthermore, at BBCH39, the concentrations of GA derivatives GA19, GA44, and GA53 are significantly reduced. Finally, at BBCH51, ABAGe concentrations are decreased, while GA concentration are increased under stress conditions.

Figure 4 illustrates the dynamic changes in phytohormone concentrations depending on environmental conditions and genotypes during plant development.

### 3.2 Comparison of Phytohormone Levels Between Genotypes Under Cold Stress

The comparison of phytohormone levels between the cold‐tolerant line SB14011 and the cold‐sensitive line SC1056 reveals significant differences in all developmental stages under both control and stress conditions (Table 3).

**TABLE 3:** The results of the *t*‐test for the difference between the two genotypes (SB14011 vs. SC1056) in terms of phytohormone concentrations including Abscisic Acid (ABA), Abscisic Acid Glucose Ester (ABAGe), Dihydrophaseic Acid (DHPA), Jasmonic acid (JA), and Phaseic Acid (PA), Gibberellins (GA), Gibberellin derivatives including GA12, GA15, GA19, GA24, GA44, and GA53 between stress and control conditions of genotype SC1056 at different developmental stages (BBCH35, BBCH39, and BBCH51); (significance level: *** 0.001; ** 0.01.; * 0.05).

Contrasts	SB14011—SC1056								
Condition	Control								
Treatment	BBCH35			BBCH39			BBCH51		
Phytohormones	df	*t*‐value	*p*	df	*t*‐value	*p*	df	*t*‐value	*p*
ABA	7.8474	−2.874	0.02112*	5.6387	1.4504	0.2002	4.5912	−1.0998	0.3257
ABAGe	6.6879	−0.8885	0.4051	/	/	/	8.3091	−0.12456	0.9038
DHPA	4.2334	−1.7092	0.1586	5.2766	1.2922	0.25	7.4214	0.51243	0.6232
PA	4.941	−0.026435	0.9799	6.7169	0.69274	0.5117	4.7095	−0.66461	0.5374
GA	5.0351	2.819	0.03686*	5.3369	−0.34405	0.744	5.2648	−1.212	0.2771
JA	6.2367	−0.27242	0.7941	3.0115	−2.0955	0.1268	8.221	−4.0755	0.003358**
JAIle	7.181	−0.25419	0.8065	3.0015	−1.7144	0.1849	7.8851	−0.47882	0.6451
GA12	4.6332	2.3627	0.0686	/	/	/	/	/	/
GA15	5	3.1399	0.02567*	/	/	/	/	/	/
GA19	4.9132	3.0798	0.02812*	6.0219	1.3928	0.2129	7.8151	−0.64674	0.5363
GA24	4	−2.1205	0.1013	/	/	/	/	/	/
GA44	6.1402	3.1056	0.02034*	6.4072	−13.306	6.61E‐06***	4.7336	−1.2661	0.2642
GA53	5.1883	2.4492	0.05621	5.6695	−0.77985	0.4668	5.2098	−1.1579	0.2972

Condition	Stress								
Treatment	BBCH35			BBCH39			BBCH51		
Pyhtohormones	df	*t*‐value	*p*	df	*t*‐value	*p*	df	*t*‐value	*p*
ABA	5.2319	−3.7407	0.01235*	8.2188	−4.9401	0.001048**	7.6644	−3.0678	0.01622*
ABAGe	7.5898	1.2013	0.2658	5	1.8845	0.1182	5	−4.3387	0.007438**
DHPA	6.5401	−1.8984	0.1024	6.8206	−0.17586	0.8655	5.2641	−1.5812	0.1717
PA	6.1319	−1.9629	0.09627	5.0985	1.5528	0.1801	9.1613	2.7674	0.02149*
GA	8.2458	0.5183	0.6179	5.733	−1.2977	0.2441	9.2204	−1.2173	0.2537
JA	5.7643	0.19537	0.8518	8.4712	0.86357	0.4116	6.1043	0.051116	0.9609
JAIle	5.8347	0.37994	0.7174	8.5983	−0.68696	0.5102	9.9298	1.3135	0.2186
GA12	7.025	1.5605	0.1625	/	/	/	/	/	/
GA15	5	2.8652	0.03519*	/	/	/	/	/	/
GA19	9.3712	−0.24661	0.8105	8.9368	−2.2459	0.05155	9.7768	−1.7846	0.1053
GA24	0.077026	6.8615	0.9408	/	/	/	5	−1	0.3632
GA44	5.6961	2.3912	0.05615	5.6619	−1.6602	0.1509	9.9307	−1.7041	0.1194

Under control conditions, the genotypes differed at BBCH35 in abscisic acid (ABA) and the gibberellins (GA) GA15, GA19, GA44, and GA53: SC1056 showed higher ABA but lower GA concentrations. At BBCH39, only GA44 differed, and at BBCH51, jasmonic acid (JA) was higher in SC1056.

Under stress conditions, significant differences in ABA were detected at all stages, with consistently higher levels in the cold‐sensitive genotype SC1056. Additionally, at BBCH35, the GA15 concentration is significantly lower in SC1056. Furthermore, at BBCH51, the ABAGe concentration is significantly higher, while the PA concentration is significantly lower in the cold‐sensitive genotype.

**FIGURE 5:**
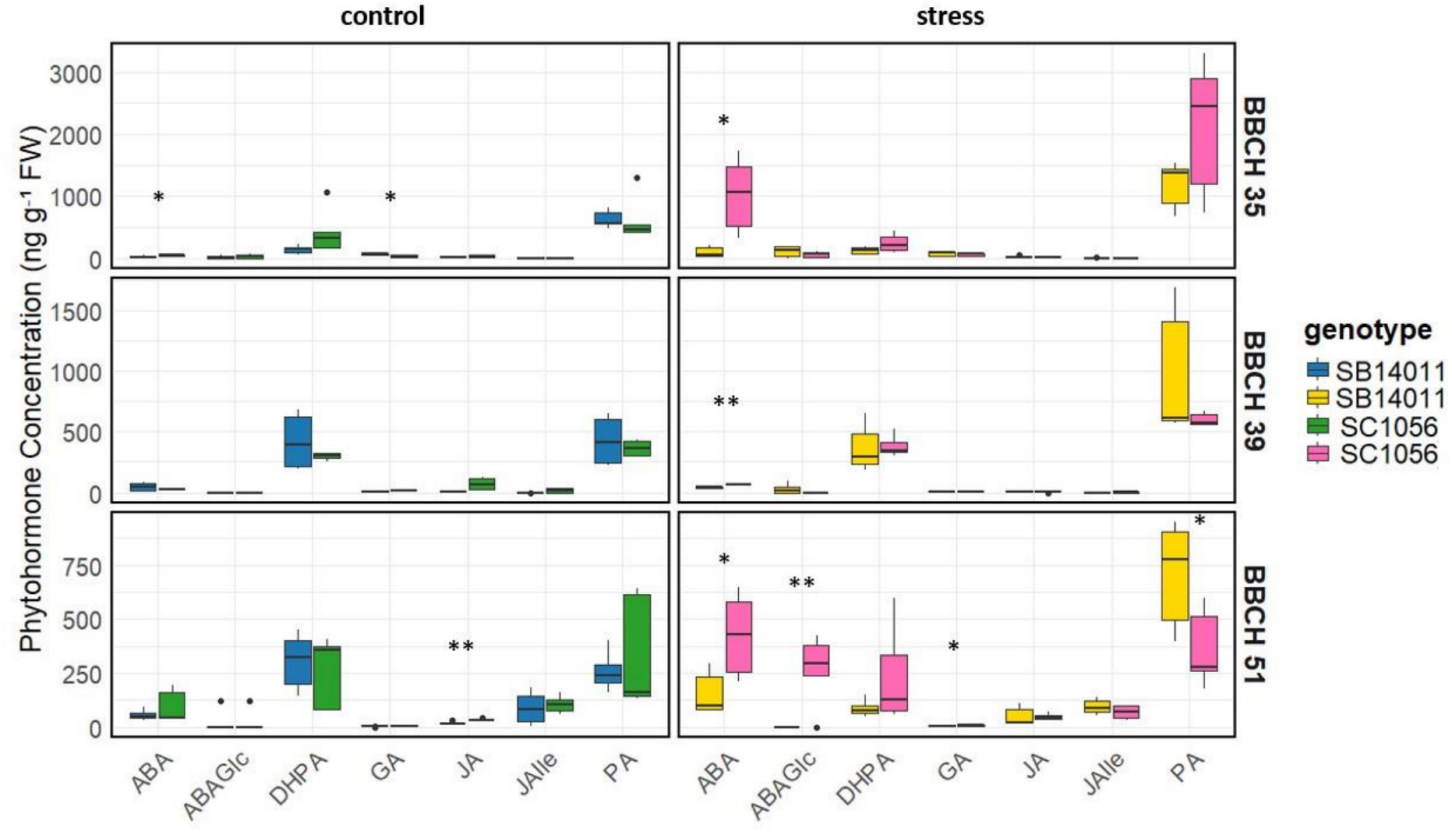
Comparison of phytohormone levels including abscisic acid (ABA), abscisic acid glucose ester (ABAGe), dihydrophaseic acid (DHPA), gibberellins (GA), jasmonic acid (JA), and phaseic acid (PA) between the cold‐tolerant genotype SB14011 and the cold‐sensitive genotype SC1056 at different developmental stages (BBCH35, BBCH39, and BBCH51) for both stress and control conditions separately.

Figure 5 illustrates these trends: ABA levels in the cold‐sensitive genotype SC1056 are markedly higher than those in the cold‐tolerant genotype SB14011 at all developmental stages. While the ABA concentration of SC1056 under stress conditions averaged 1019.3 at BBCH35, 66.45 at BBCH39, and 424.8 at BBCH51, SB14011 exhibited values of only 94.41, 41.11, and 152.86, respectively. This trend is visually represented in Figure 3. Considering all phytohormones collectively, it is noticeable that the phytohormone level after cold stress in BBCH35 of the cold‐sensitive genotype, exceeding 2000, is significantly higher than that of the cold‐tolerant genotype. Additionally, for both genotypes, the overall phytohormone level is higher under stress conditions compared to control conditions.

**FIGURE 6:**
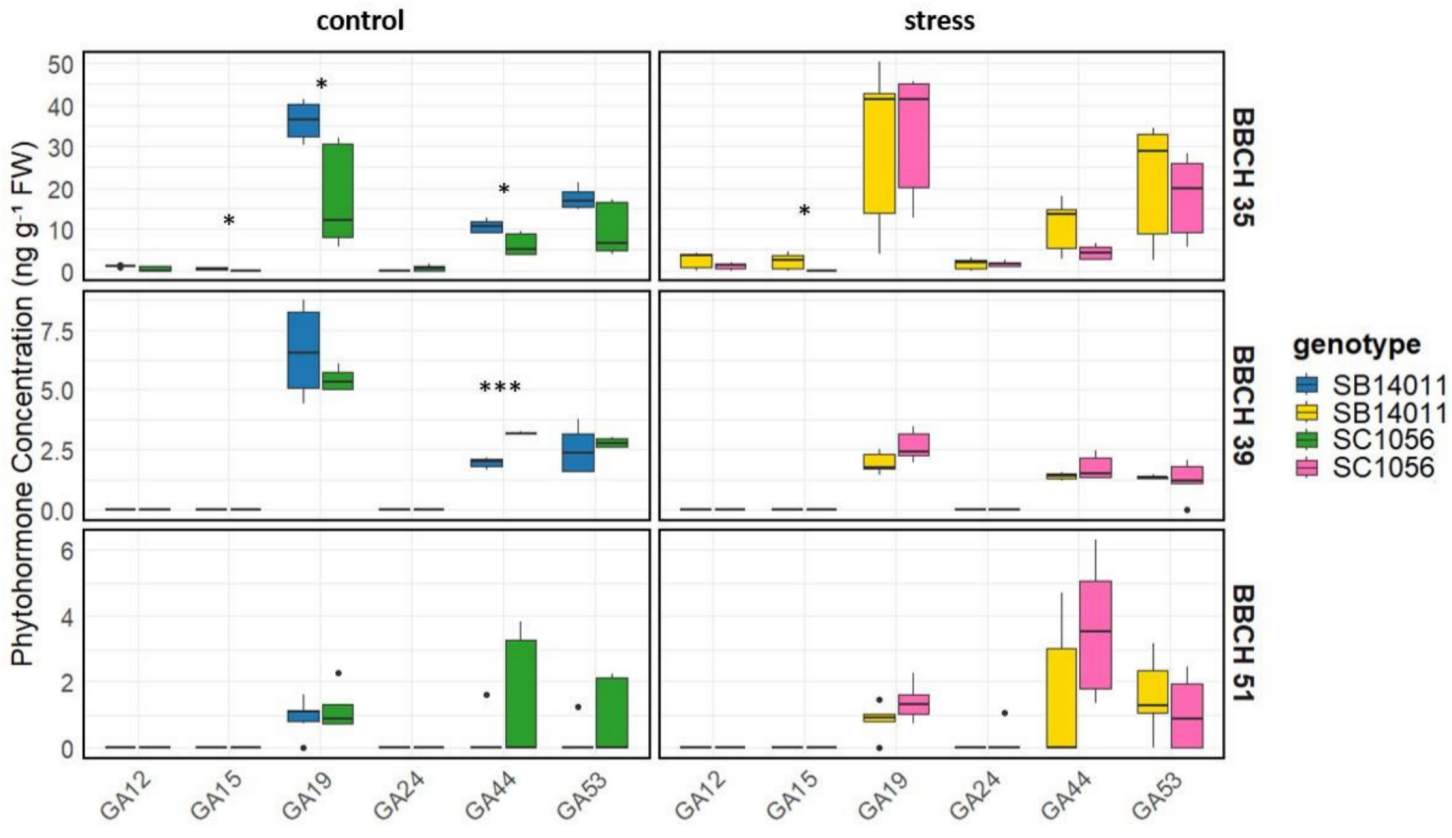
Comparison of phytohormone levels of gibberellin derivatives including GA12, GA15, GA19, GA24, GA44, and GA53 between the cold‐tolerant genotype SB14011 and the cold‐sensitive genotype SC1056 at different developmental stages (BBCH35, BBCH39, and BBCH51), separately for stress and control conditions of.

Figure 6 depicts the phytohormone levels of individual gibberellin derivatives in the two genotypes SB14011 and SC1056 under stress and control conditions. It can be observed that under stress conditions, the concentration of GA15 at BBCH35 is detectable only in the cold‐tolerant genotype SB14011 (2.227 ng), while it is absent in SC1056.

### 3.3 Differences in Phytohormone Levels Between Developmental Stages

#### 3.3.1 Abscisic Acid and Abscisic Acid Glucose Ester

No significant differences in abscisic acid (ABA) and abscisic acid glucose ester (ABAGe) levels were observed among the investigated developmental stages for both the cold‐tolerant and the cold‐sensitive genotypes under control conditions (Table 4). While in SB14011, the ABA concentration did not differ among developmental stages even under stress conditions, SC1056 exhibited significant differences between developmental stages BBCH35 and BBCH39, as well as between BBCH35 and BBCH51.

**TABLE 4:** Results of the ANOVA and subsequent Tukey test examining the phytohormone concentration of abscisic acid (ABA) and abscisic acid glucose ester (ABAGe) at various developmental stages under control and stress conditions for the cold‐tolerant genotype SB14011 and the cold‐sensitive genotype SC1056 (significance level: *** 0.001; ** 0.01.; *0.05).

SB14011	ANOVA			ABA					ABAGe		
		*p* = 0.143					*p* = 0.491				
Condition	Contrasts	Estimate	SE	df	t.ratio	*p*	Estimate	SE	df	t.ratio	*p*

BBCH35‐BBCH39	/	/	/	/	/	/	/	/	/	/
Control	BBCH35‐BBCH51	/	/	/	/	/	/	/	/	/	/
	BBCH39‐BBCH51	/	/	/	/	/	/	/	/	/	/
SC1056	ANOVA	*p* = 0.133					*p* = 0.463				
	BBCH35‐BBCH39	/	/	/	/	/	/	/	/	/	/
Control	BBCH35‐BBCH51	/	/	/	/	/	/	/	/	/	/
	BBCH39‐BBCH51	/	/	/	/	/	/	/	/	/	/
SB14011	ANOVA	*p* = 0.0824					*p* = 0.0135 *				
	BBCH35‐BBCH39	/	/	/	/	/	77.6	32.2	15	2.406	0.0295
Stress	BBCH35‐BBCH51	/	/	/	/	/	106.3	32.2	15	3.297	0.0049
	BBCH39‐BBCH51	/	/	/	/	/	28.7	32.2	15	0.891	0.3872
SC1056	ANOVA	*p* = 0.00138 **					*p* = 0.000328 ***				
	BBCH35‐BBCH39	953	210	15	4.548	0.0004	56.8	53.5	15	1.061	0.3055
Stress	BBCH35‐BBCH51	594	210	15	2.837	0.0125	−215.2	53.5	15	−4.019	0.0011
	BBCH39‐BBCH51	358	210	15	−1.71	0.1078	−272	53.5	15	−5.08	0.0001

For ABAGe, both genotypes showed stage‐dependent differences: SB14011 between BBCH35 and BBCH39 and BBCH35 and BBCH51, and SC1056 between BBCH35 and BBCH51 and BBCH39 and BBCH51.

**FIGURE 7:**
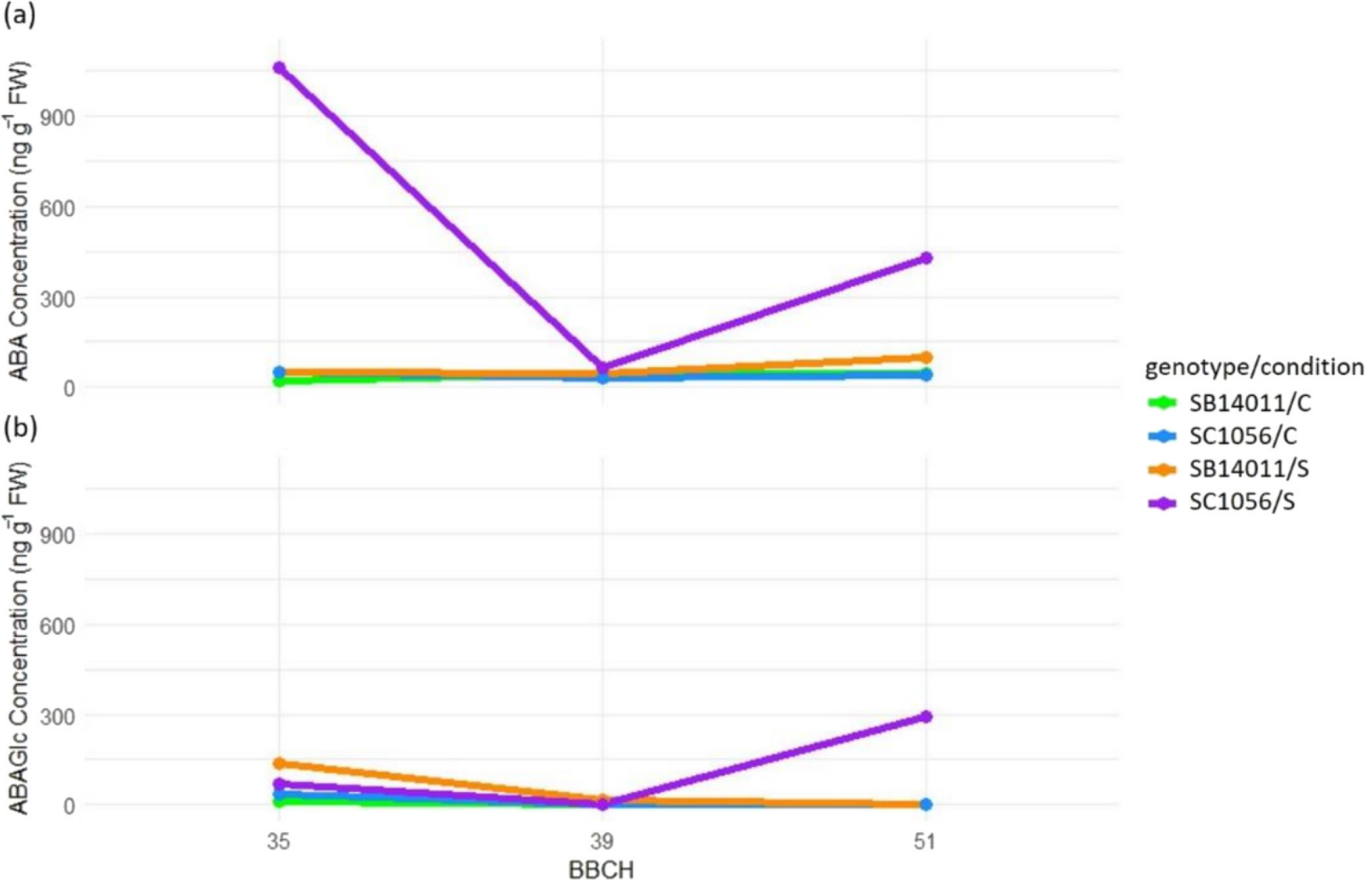
Representation of genotype x condition x treatment interaction for abscisic acid (ABA) and abscisic acid glucose ester (ABAGe) concentration; treatment: BBCH35, BBCH39 and BBCH51; genotypes: the cold‐tolerant genotype SB14011 and the cold‐sensitive genotype SC1056, conditions: optimal conditions (C), stress (S).

When examining the ABA concentrations of the cold‐sensitive genotype SC1056 in Figure 7a under stress conditions at different developmental stages (BBCH35, BBCH39, and BBCH51), significant differences become apparent. Particularly, the significantly higher ABA concentrations following cold stress from BBCH35 onwards are mentionable. Under control conditions and for the cold‐tolerant genotype SB14011, even under stress conditions, ABA concentrations consistently remain below 100 ng g^−1^ FW. In Figure 7b, the significantly higher ABAGe concentration following cold stress in SC1056 at BBCH51 and in SB14011 at BBCH35 compared to other treatments within each genotype is clearly evident.

#### 3.3.2 Dihydrophaseic Acid and Phaseic Acid

When comparing the phytohormone levels of dihydrophaseic acid (DHPA), significant differences among developmental stages were observed only under control conditions in the cold‐tolerant genotype SB14011 (Table 5), specifically between BBCH35 and BBCH39. No significant differences were observed in the cold‐sensitive genotype SC1056 under control conditions. Under stress, DHPA levels remained constant across stages in both genotypes.

**TABLE 5:** Results of the ANOVA and subsequent Tukey test examining the phytohormone concentration of dihydrophaseic acid (DHPA) and phaseic acid (PA) at various developmental stages under control and stress conditions for the cold‐tolerant genotype SB14011 and the cold‐sensitive genotype SC1056 (significance level: *** 0.001; ** 0.01.; *0.05).

SB14011	ANOVA			DHPA					PA		
		*p* = 0.0218 *					*p* = 0.00291				
Condition	Contrasts	Estimate	SE	df	t.ratio	*p*	Estimate	SE	df	t.ratio	*p*
Control	BBCH35‐BBCH39	−285	90.7	15	−3.142	0.0067	/	/	/	/	/
Control	BBCH35‐BBCH51	−168	90.7	15	−1.849	0.0842	/	/	/	/	/
Control	BBCH39‐BBCH51	117	90.7	15	1.292	0.2158	/	/	/	/	/
SC1056	ANOVA	*p* = 0.55					*p* = 0.239				
Control	BBCH35‐BBCH39	/	/	/	/	/	/	/	/	/	/
Control	BBCH35‐BBCH51	/	/	/	/	/	/	/	/	/	/
Control	BBCH39‐BBCH51	/	/	/	/	/	/	/	/	/	/
SB14011	ANOVA	0.00133					0.171				
Stress	BBCH35‐BBCH39	/	/	/	/	/	/	/	/	/	/
Stress	BBCH35‐BBCH51	/	/	/	/	/	/	/	/	/	/
Stress	BBCH39‐BBCH51	/	/	/	/	/	/	/	/	/	/
SC1056	ANOVA	0.221					*p* = 0.00053 ***				
Stress	BBCH35‐BBCH39	/	/	/	/	/	1524	375	15	4.066	0.001
Stress	BBCH35‐BBCH51	/	/	/	/	/	1761	375	15	4.699	0.0003
Stress	BBCH39‐BBCH51	/	/	/	/	/	237	375	15	0.633	0.5363

Regarding the phaseic acid (PA) levels, SB14011 exhibited no significant differences between developmental stages under either control and stress conditions. SC1056 only showed significant differences under stress conditions between BBCH35 and BBCH39, as well as BBCH35 and BBCH51.

**FIGURE 8:**
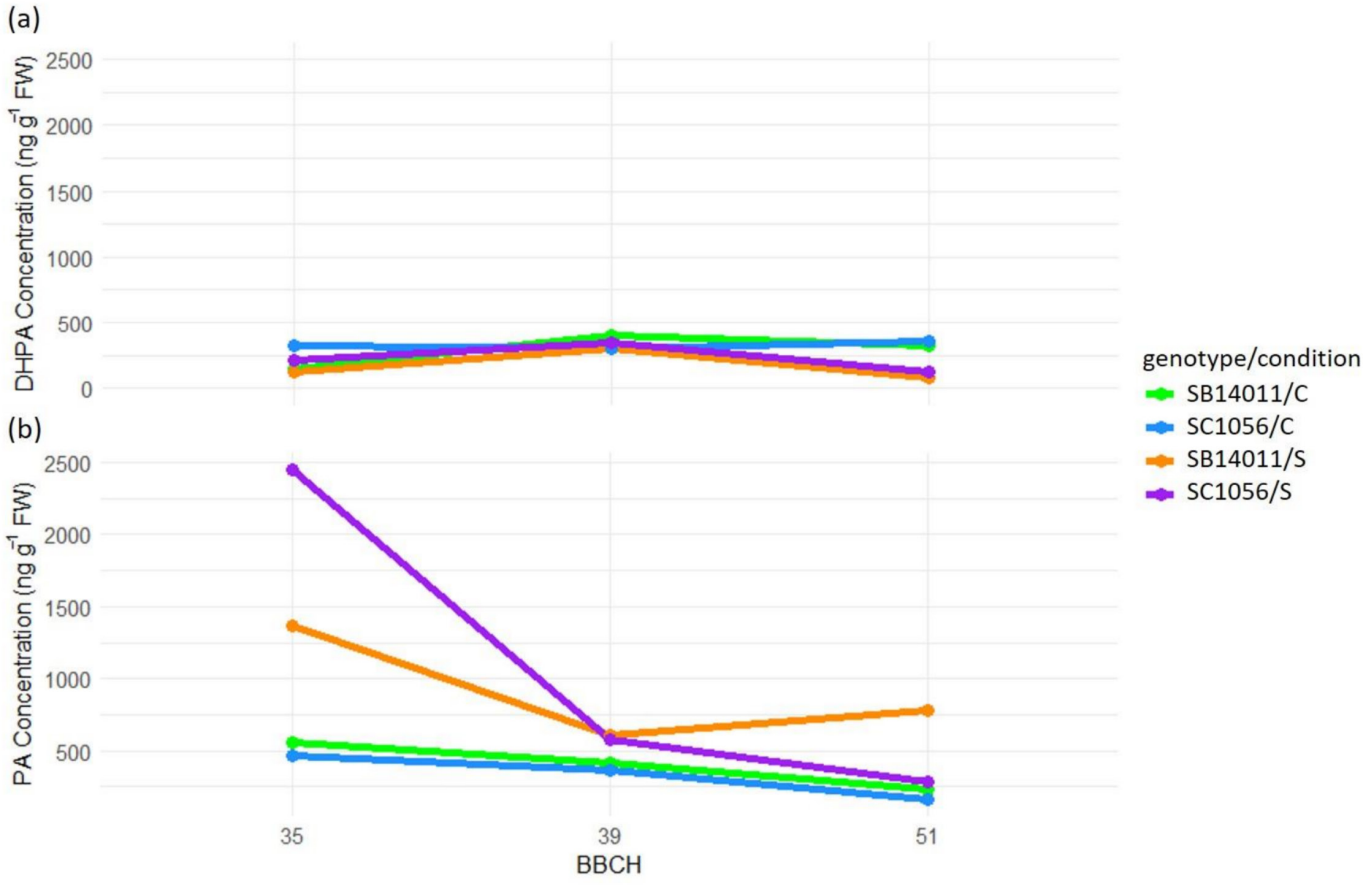
Representation of genotype x condition x treatment interaction for DHPA and PA concentration; treatment: BBCH35, BBCH39 and BBCH51; genotypes: the cold‐tolerant genotype SB14011 and the cold‐sensitive genotype SC1056, condition: optimal conditions (C), stress (S).

When considering the DHPA and PA concentrations in the cold‐sensitive genotype SC1056 (Figure 8), no fluctuations between developmental stages are observed under optimal conditions. However, in the cold‐tolerant genotype SB14011, Figure 6 clearly illustrates that DHPA accumulation is significantly lower in BBCH35 compared to other developmental stages (BBCH39 and BBCH51). Regarding PA, no variations between stages can be foundin SB14011. However, in both genotypes, a continuous decline in PA concentration from BBCH35 to BBCH51 is observed. Particularly noteworthy is the significantly increased concentration of almost 2500 ng g^−1^ FW of the SC1056 genotype after cold stress at BBCH35, which is remarkably higher than that of the other two treatment levels, BBCH39 and BBCH51.

#### 3.3.3 Jasmonic Acid and JA‐Isoleucine

When comparing the phytohormone levels of jasmonic acid (JA), significant differences between developmental stages were observed only under stress conditions for the cold‐sensitive genotype SC1056 (see Table 6). Here, significant differences were noted between developmental stages BBCH35 and BBCH51, as well as BBCH39 and BBCH51.

**TABLE 6:** Results of the ANOVA and subsequent Tukey test examining the phytohormone concentration of jasmonic acid (JA) and JA‐isoleucine (JAIle) at various developmental stages under control and stress conditions for the cold‐tolerant genotype SB14011 and the cold‐sensitive genotype SC1056 (Significance level: *** 0.001; ** 0.01.; *0.05).

				JA					JAIle		
SB14011	ANOVA	*p* = 0.274					*p* = 0.00509 **				
Condition	Contrasts	Estimate	SE	df	t.ratio	*p*	Estimate	SE	df	t.ratio	*p*
Control	BBCH35‐BBCH39	/	/	/	/	/	1.73	25.3	15	0.068	0.9465
Control	BBCH35‐BBCH51	/	/	/	/	/	−84.77	25.3	15	−3.356	0.0043
Control	BBCH39‐BBCH51	/	/	/	/	/	−86.49	25.3	15	−3.424	0.0038
SC1056	ANOVA	*p* = 0.135					*p* = 0.000202 ***				
Control	BBCH35‐BBCH39	/	/	/	/	/	−15.4	18	11	−0.855	0.4107
Control	BBCH35‐BBCH51	/	/	/	/	/	−101.5	17	11	−5.977	0.0001
Control	BBCH39‐BBCH51	/	/	/	/	/	−86.1	18	11	−4.78	0.0006
SB14011	ANOVA	0.0718					5.09e‐07 ***				
Stress	BBCH35‐BBCH39	/	/	/	/	/	−0.727	11.4	15	−0.064	0.9502
Stress	BBCH35‐BBCH51	/	/	/	/	/	−93.578	11.4	15	−8.182	< 0.0001
Stress	BBCH39‐BBCH51	/	/	/	/	/	−92.852	11.4	15	−8.118	< 0.0001
SC1056	ANOVA	*p* = 5.69e‐06 ***					*p* = 8.96e‐06 ***				
Stress	BBCH35‐BBCH39	3.58	5.57	15	0.641	0.531	−2.04	10.5	15	−0.194	0.8489
Stress	BBCH35‐BBCH51	−35.5	5.57	15	−6.368	< 0.0001	−69	10.5	15	−6.555	< 0.0001
Stress	BBCH39‐BBCH51	−39.07	5.57	15	−7.01	< 0.0001	−66.96	10.5	15	−6.361	< 0.0001

In contrast, developmental stages showed significant differences in terms of jasmonic acid‐isoleucine (JA‐Ile) for both genotypes under both control and stress conditions. Both SB14011 and SC1056 exhibited significant differences between developmental stages BBCH35 and BBCH39, as well as between BBCH35 and BBCH51 in both control and stress conditions.

**FIGURE 9:**
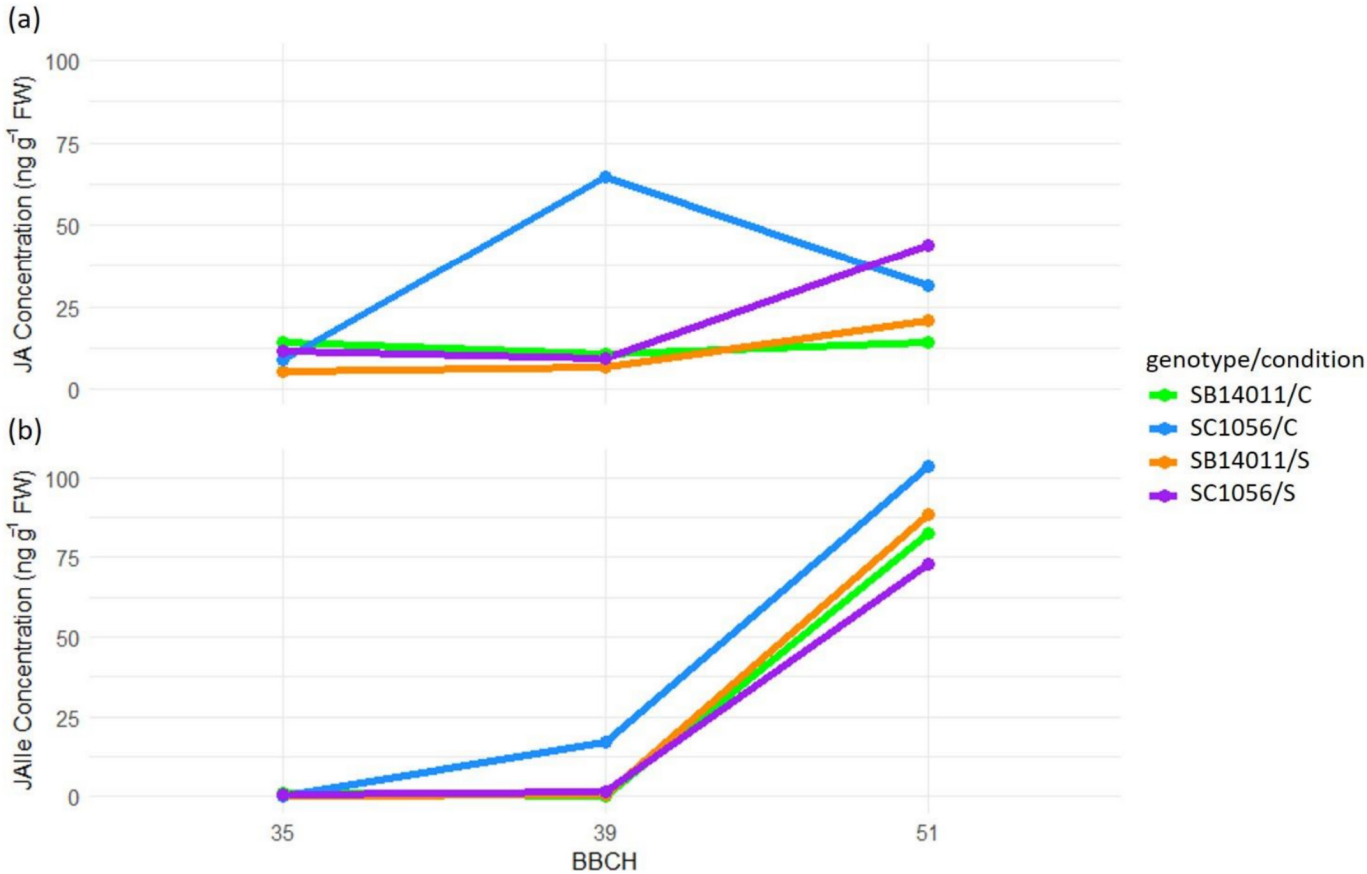
Representation of genotype x condition x treatment interaction for JA and JAIle concentration; treatment: BBCH35, BBCH39 and BBCH51; genotypes: the cold‐tolerant genotype SB14011 and the cold‐sensitive genotype SC1056, condition: optimal conditions (C), stress (S).

In Figure 9a, the cold‐sensitive genotype SC1056 shows a pronounced increase in JA concentration after cold stress at BBCH51, whereas the increase observed under optimal conditions at BBCH39 is not significant compared to the other stages. Figure 9b shows that JA‐Ile concentrations steadily increase from BBCH35 to BBCH51 in both genotypes, regardless of conditions, with the highest accumulation at BBCH51. This stage differs significantly from BBCH35 and BBCH39, which show similar concentrations.

#### 3.3.4 Gibberellins

Examination of the phytohormone levels of gibberellins (GA12, GA15, GA19, GA24, GA44, and GA53) revealed no significant differences between developmental stages under control conditions for the cold‐tolerant genotype. However, under stress conditions, significant differences were observed between developmental stages BBCH35 and BBCH39, as well as BBCH35 and BBCH51 (Table 7).

**TABLE 7:** Results of the ANOVA analysis and subsequent Tukey test examining the phytohormone concentration of gibberellins (GA) at various developmental stages under control and stress conditions for the cold‐tolerant genotype SB14011 and the cold‐sensitive genotype SC1056 (significance level: *** 0.001; ** 0.01.; *0.05).

				GA		
SB14011	ANOVA	*p* = 4.94e‐12 ***				
Condition	Contrasts	Estimate	SE	df	t.ratio	*p*
Control	BBCH35‐BBCH39	54.53	3.2	15	17.045	< 0.0001
Control	BBCH35‐BBCH51	64.14	3.2	15	20.047	< 0.0001
Control	BBCH39‐BBCH51	9.61	3.2	15	3.002	0.0089
SC1056	ANOVA	*p* = 0.0128 *				
Control	BBCH35‐BBCH39	23.33	9.41	11	2.480	0.0306
Control	BBCH35‐BBCH51	31.28	8.87	11	3.526	0.0048
Control	BBCH39‐BBCH51	7.95	9.41	11	0.844	0.4165
SB14011	ANOVA	*p* = 0.00084 ***				
Condition	Contrasts	Estimate	SE	df	t.ratio	*p*
Stress	BBCH35‐BBCH39	65.252	15.6	15	4.177	0.0008
Stress	BBCH35‐BBCH51	6.077	15.6	15	4.230	0.0007
Stress	BBCH39‐BBCH51	0.825	15.6	15	0.053	0.9586
SC1056	ANOVA	*p* = 5.46e‐05 ***				
Stress	BBCH35‐BBCH39	52.72	9.52	15	5.540	0.0001
Stress	BBCH35‐BBCH51	52.21	9.52	15	5.486	0.0001
Stress	BBCH39‐BBCH51	−0.51	9.52	15	−0.054	0.9580

The cold‐sensitive genotype SC1056 exhibited significant differences between developmental stages under both control and stress conditions. In both cases, BBCH35 differed from BBCH39 and BBCH51.

**FIGURE 10:**
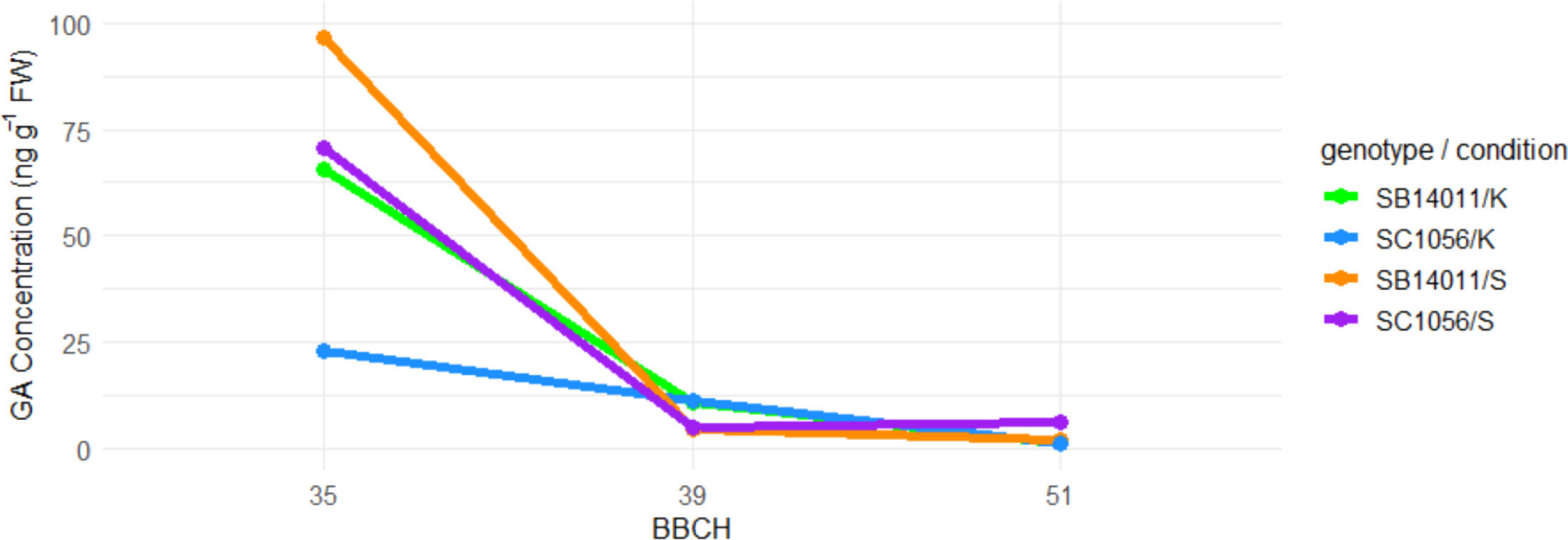
Representation of genotype x condition x treatment interaction for the gibberellin concentration; treatment: BBCH35, BBCH39 and BBCH51; genotypes: the cold‐tolerant genotype SB14011 and the cold‐sensitive genotype SC1056, condition: optimal conditions (C), stress (S).

It is evident that in both genotypes, regardless of the condition, GA concentration consistently decreases from the early developmental stage BBCH35 through BBCH39 to the later BBCH51 (Figure 10). The highest accumulation is observed in BBCH35 for the cold‐tolerant genotype under stress conditions. Interestingly, the GA concentration of the cold‐sensitive genotype under stress conditions is comparable to that of the cold‐tolerant genotype under optimal conditions.

### 3.4 Correlation Analysis of Phytohormones, Pollen Traits, and Yield Parameters

**FIGURE 11:**
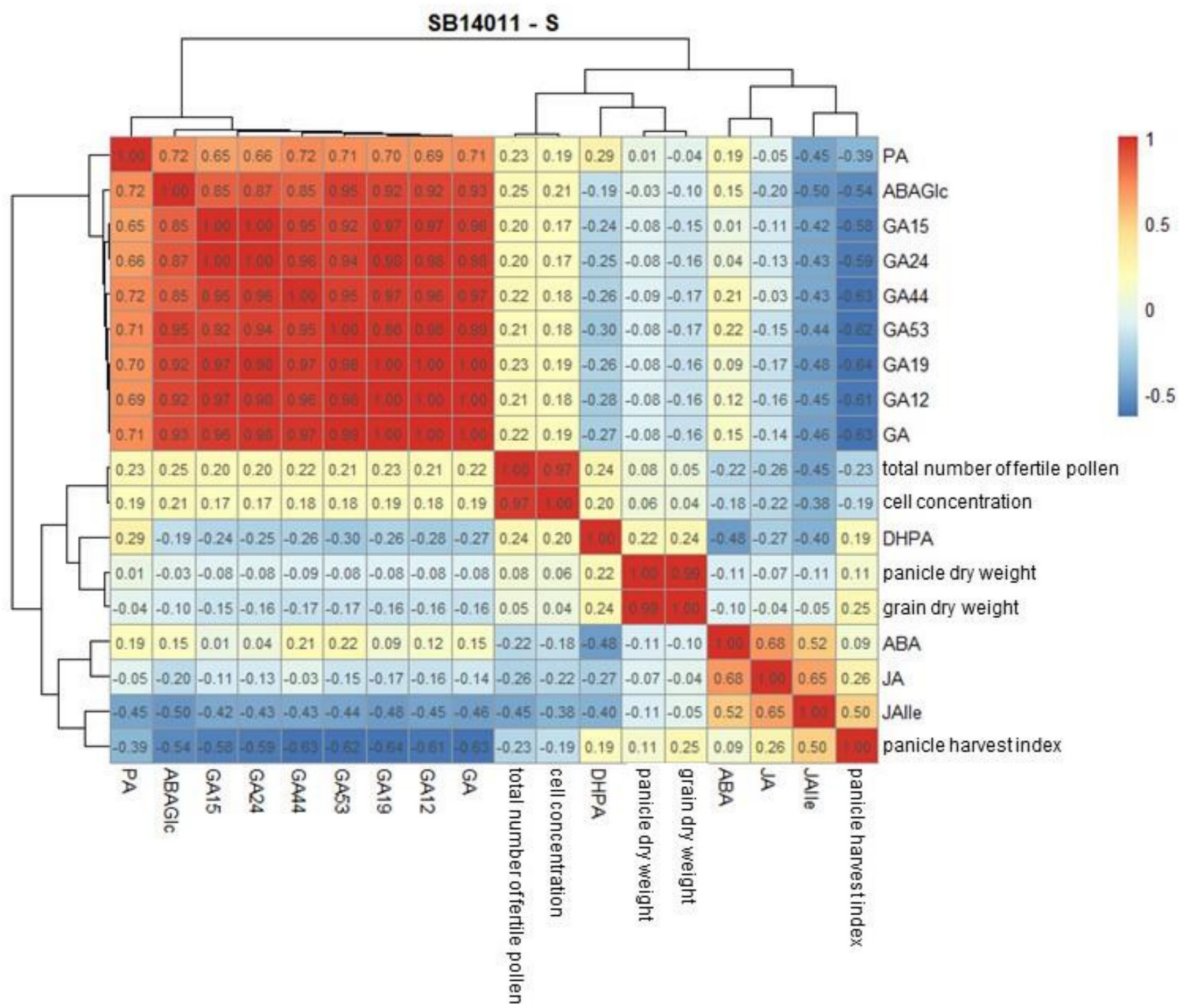
Heatmap illustrating Pearson's correlations among the analyzed traits for the cold‐tolerant genotype SB14011 under cold stress: abscisic acid (ABA), abscisic acid glucose ester (ABAGe), dihydrophaseic acid (DHPA), jasmonic acid (JA), phaseic acid (PA), gibberellins (GA) and their derivatives (GA12, GA15, GA19, GA24, GA44, and GA53), total number of fertile pollen, cell concentration, panicle harvest index, panicle dry weight, and grain dry weight. Strong positive correlations (r = 1) are shown in red, while strong negative correlations (r = −1) are shown in blue.

**FIGURE 12:**
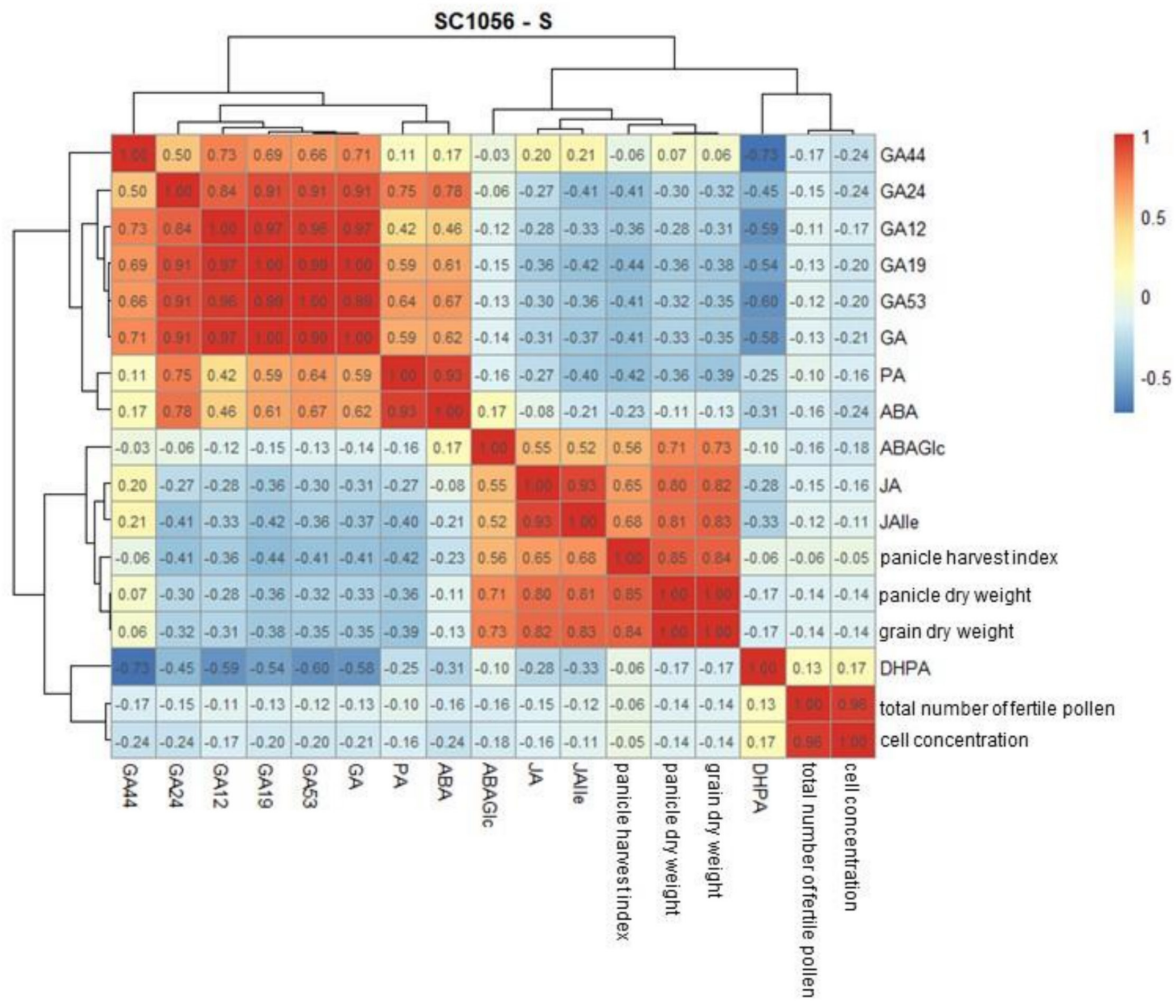
Heatmap illustrating Pearson's correlations among the analyzed traits for the cold‐sensitive genotype SC1056 under cold stress: abscisic acid (ABA), abscisic acid glucose ester (ABAGe), dihydrophaseic acid (DHPA), jasmonic acid (JA), phaseic acid (PA), gibberellins (GA) and their derivatives (GA12, GA15, GA19, GA24, GA44, and GA53), total number of fertile pollen, cell concentration, panicle harvest index, panicle dry weight, and grain dry weight. Strong positive correlations (r = 1) are shown in red, while strong negative correlations (r = −1) are shown in blue.

The Pearson correlation was calculated to examine possible relationships between phytohormones, pollen traits, and yield parameters. The yield and pollen data were taken from the dataset of Neitzert et al. (2025). The results for both genotypes under stress conditions are shown in Figure 11 and Figure 12 (correlations under optimal conditions are provided in the Supplementary section, Figure S1, S2). The following section describes only those correlations between yield, pollen traits, and phytohormones that are relevant for interpreting the cold stress response. Yield parameters include grain dry yield, panicle dry yield, and panicle harvest index, while pollen traits include the total number of fertile pollen and cell concentration.

#### 3.4.1 SB14011 (Cold‐Tolerant)

Under stress conditions (Figure 11), the panicle harvest index (PHI) shows a strong negative correlation with all gibberellins (GA12, GA15, GA19, GA24, GA44, and GA53). Apart from that, no strong correlations were found between phytohormones and either yield or pollen traits.

Under optimal conditions, neither yield parameters nor pollen traits nor phytohormones were strongly correlated with each other (Figure S1).

#### 3.4.2 SC1056 (Cold‐Sensitive)

Under stress conditions (Figure 12), yield parameters were strongly correlated with ABAGlc, JA, and JAIle. Pollen traits, however, showed no strong correlations with either yield parameters or phytohormones.

Under optimal conditions, pollen traits as well as JAIle were strongly negatively correlated with GA44, GA19, GA53, and total GA. A strong positive correlation was found between pollen traits and JAIle. Yield parameters showed no strong correlations with either pollen traits or phytohormones (Figure S2).

## 4 Discussion

### 4.1 Abscisic Acid and Its Derivatives

#### 4.1.1 Abscisic Acid

The presence of genes regulating the concentration of abscisic acid (ABA) and enabling cold‐tolerant genotypes to produce lower amounts of ABA has been previously reported for rice (Sharma and Nayyar 2016). The elevated synthesis of ABA has been attributed to increased expression of the two biosynthetic ABA genes, *OSZEP1* and *OSNCED3*. When comparing with the cold‐sensitive rice genotype, the cold‐tolerant rice genotype exhibited lower expression of both genes, leading to the observed reduced ABA levels (Oliver et al. 2007; Ji et al. 2011). Our results confirm this phenomenon for sorghum as well. Here, the cold‐tolerant inbred line SB14011 maintains a consistently low level of ABA under cold stress across all stages when compared to optimal growth conditions. Conversely, a significant increase in ABA concentration under cold stress at all stages (BBCH35, BBCH39, and BBCH51) can be confirmed in the cold‐sensitive genotype SC1056. This could indicate that the ABA regulatory mechanisms observed in rice may also be transferable to sorghum. In rice, the presence of genetic sequences in the cold‐tolerant line enables it to maintain a low ABA concentration, whereas the absence of such sequences in the cold‐sensitive genotype leads to elevated ABA levels (Ji et al. 2011; Sharma and Nayyar 2016).

Pollen sterility has been discussed in several studies as the main reason for reduced cereal yields after cold stress. Various studies have shown that ABA levels can directly influence tapetal development and thus pollen fertility (Sakata et al. 2014). Sharma and Nayyar suggest that the ability to accumulate low ABA levels under cold stress contributes to making anthers more resistant to ensure the development of viable pollen. Increased ABA accumulation as a cause of impaired pollen fertility has been proposed in several studies. In sorghum and rice, meiosis and microspore together with tapetal development leading to male sterile pollen have been identified as particularly cold‐sensitive (Downes and Marshall 1971; Mamun et al. 2006; Wood et al. 2006; Gothandam et al. 2007). Cold‐tolerant rice plants are capable of further reducing ABA levels in the anthers through increased expression of ABA hydroxylation genes (Nambara and Marion‐Poll 2005; Oliver et al. 2007). Here, ABA is converted into phaseic acid, thereby promoting ABA degradation. In contrast to the ABA genes, the expression of the two ABA 8‐hydroxylase genes, *ABA8ox1* and *ABA8ox2*, is higher in cold‐tolerant genotypes (Oliver et al. 2007; Li et al. 2011). Besides the ABA hydroxylation genes, the cell wall invertase gene also appears to play a key role in rice. In the tapetum, in addition to maintaining low ABA levels, the expression of the cell wall invertase gene (*INV4*) ensures the development of fertile pollen (Ji et al. 2011). In cold‐sensitive genotypes, the suppression of the cell wall invertase gene by increased ABA accumulation appears to be the cause of abnormal pollen development. However, this mechanism does not seem to apply to all examined developmental stages in sorghum.

The cold‐sensitive genotype used in a previous study conducted by us showed no significant reduction in pollen fertility at the cold‐sensitive developmental stage BBCH39. Despite cold influence at this stage, pollen fertility remained at a level comparable to that of the control and post‐cold stage BBCH51. Only cold stress from BBCH35 resulted in a significant reduction in fertile pollen, yet pollen fertility still exceeded 60%. A pollination experiment revealed that pollen fertility was not the determining factor for yield losses caused by cold stress (Neitzert et al. 2025). This conclusion is supported by the correlation analysis, which revealed no significant relationships between pollen traits and yield parameters—neither under optimal nor under stress conditions. This underscores that the observed yield losses are not primarily attributable to reduced pollen fertility. Based on this, it can be concluded that the significantly increased ABA concentration after cold stress at BBCH39 is not accompanied by a reduction in pollen fertility. Therefore, the theory that the ability to develop fertile pollen depends on the genotype's ability to maintain low ABA concentrations can only be applied for cold from BBCH35 onwards. As described in other cereal species, ABA 8′‐hydroxylase and cell wall invertase may play an important role in controlling ABA homeostasis in the anthers and pollen development during the reproductive stage (Ji et al. 2011).

Contrary to reports in rice and other cereals, one explanation for the typically high pollen fertility despite elevated ABA accumulation after cold stress at BBCH39 in the cold‐sensitive genotype could be impairment of the female floral organ. Impairment of the female floral organ can also be discussed for cold stress at BBCH35, as increased cold sensitivity of the female floral organ has already been observed in other plant species, leading to various impairments, such as irreversible abortion of the egg cell or delayed maturation and increased embryo mortality (Casper 1990; Srinivasan et al. 1999). Since the functionality of the egg cell is crucial for normal pistil operation (Dumas et al. 1984), this could indicate that cold also affects this function in sorghum. Further studies are required to precisely determine to what extent and in which phases of female gametogenesis the effects are most affected. Thus, sorghum would fundamentally differ from rice, where low ABA levels in the anthers are considered a key determinant of fertility. At the BBCH51 stage, cereals are in the transition between spikelet and floret development (Guo et al. 2015). Schaffasz et al. (2019) showed that sorghum is no longer susceptible to cold stress from BBCH stage 43 onward, as indicated by high pollen viability of over 80%. The resilience of the cold‐tolerant genotype SB14011 after cold stress at the BBCH51 stage could be explained by the completed and more sensitive spikelet development. Cold stress and the resulting higher ABA levels appear to have no further impact on the development of the male or female floral organ at the onset of floret development, which aligns with the findings of Schaffasz et al. (2019).

When considering the cold‐tolerant genotype, the ABA level remains consistently high across all developmental stages, but never at zero. This is because a certain amount of ABA is required for normal anther development and the ensuring of fertile pollen (Wang et al. 1999). For sorghum, the average ABA concentration in the cold‐tolerant genotype after cold stress is 96.127 ng g^−1^ FW across all stages examined. In comparison, the average ABA concentration of the cold‐sensitive genotype is five‐fold higher (503.52 ng g^−1^ FW).

#### 4.1.2 Abscisic Acid Glucose Ester (ABAGe)

Looking at abscisic acid glucose ester (ABAGe), a conjugate of abscisic acid serving as the storage and transport form of the hormone ABA, no significant differences in ABAGe concentration between control and stress conditions at sensitive stages (BBCH35 and BBCH39) could be observed in either the cold‐tolerant or cold‐sensitive genotype. Furthermore, the ABAGe concentration of both genotypes remains at a similar level during sensitive stages. When comparing individual developmental stages within each genotype, significant differences occur under cold stress. While SB14011 (cold‐tolerant) exhibits significantly higher ABAGe concentrations in BBCH35 compared to BBCH51 after cold stress, SC1056 (cold‐sensitive) shows significantly lower ABAGe concentrations in BBCH35 and BBCH39 compared to BBCH51. The amount of ABA accumulating in a plant is influenced not only by ABA production but also by processes such as ABA degradation and conjugation to other molecules (Ren et al. 2007). The observed increased accumulation of ABAGe in BBCH35 could be due to ABA regulation through conjugation. At the same time, the reduced amount of ABAGe in the sensitive developmental stages of the cold‐sensitive genotype could be explained as a consequence of increased ABA release. Especially in BBCH35, where ABA peaked at 1729.8 ng g^−1^ FW after cold stress, a depleted ABAGe reservoir seems plausible. Under optimal growth conditions, the ABAGe levels of both genotypes remain consistent across all developmental stages, reinforcing the theory of the depleted ABAGe reservoir in SC1056 after cold exposure. The significant correlation of ABAGlc with yield parameters in the cold‐sensitive genotype (SC1056) under cold stress indicates that the glucoside form of the hormone is directly linked to yield performance.

#### 4.1.3 Dihydrophaseic Acid and Phaseic Acid

Besides the previous inactivation of abscisic acid (ABA) through conjugation, ABA can also be deactivated through oxidation (Zeevaart 1999; Kushiro et al. 2004; Nambara and Marion‐Poll 2005). In this oxidation process, phaseic acid (PA) and dihydrophaseic acid (DHPA) are breakdown products of ABA. The most common hydroxylation in ABA catabolism occurs at C‐8′, generating 8’‐Hydroxy‐ABA, which later isomerizes to PA. DHPA is produced through further reduction of PA. At this point, alongside DHPA, the analog epi‐DHPA can also form (Krochko et al. 1998; Cutler and Krochko 1999). In cold‐tolerant rice, it has been demonstrated that the expression of ABA 8‐hydroxylase genes, which convert ABA to phaseic acid, was significantly higher than in cold‐sensitive varieties (Oliver et al. 2007; Ji et al. 2011).

In this study, after cold stress, PA is elevated in both tolerant and susceptible genotypes. This implies that the expression of ABA 8‐hydroxylase genes seems to be high regardless of genotype‐specific cold tolerance levels. While the comparison between growth under optimal conditions and under cold stress reveals that the cold‐tolerant line exhibits only an increased PA concentration in BBCH35, the PA concentration is significantly elevated in both sensitive stages (BBCH35 and BBCH39) in the cold‐sensitive genotype.

Therefore, the cold‐sensitive genotype appears to utilize oxidation instead of conjugation (see Section 4.1.2) to counteract the significantly increased ABA concentrations in stages sensitive to cold. The elevated ABAGe (Section 4.1.2) and PA levels in the cold‐tolerant genotype suggest that it utilizes both conjugation and oxidation pathways to deactivate ABA. This indicates that oxidation and conjugation pathways are active under cold stress. However, while SB14011 shows consistent PA concentrations between developmental stages after cold stress, SC1056 exhibits significantly higher PA levels in BBCH35 compared to BBCH39 and BBCH51. This can be explained by the extremely high accumulation of ABA in this developmental stage. The generally high PA contents compared to ABA and DHPA suggest that PA has a long half‐life and is metabolized relatively slowly to DHPA.

In contrast, in other plant species, degradation of ABA predominates over PA or DHPA (Balsevich et al. 1994; Setha et al. 2005; Huang et al. 2008). These findings suggest that, as described by Garbero et al. (2011), ABA metabolism is regulated differently in various plant species. The data suggest that sorghum maintains ABA homeostasis through concurrently active conjugation (ABAGe) and oxidation pathways (PA/DHPA). In contrast to rice, where increased ABA oxidation to PA is associated with cold tolerance, both genotypes examined here show comparable PA accumulation. This indicates that the activity/expression of ABA 8‐hydroxylase genes in sorghum is not a genotype‐specific limiting factor—at least in the genotypes, developmental stages, and conditions tested.

### 4.2 Gibberellins

Cold stress in rice causes a reduction in the endogenous levels of bioactive gibberellins. Cold‐tolerant rice genotypes capable of maintaining a sufficient pool of bioactive gibberellins under cold stress through increased gibberellins (GA) catabolism have been identified (Sharma and Nayyar 2016). Problems arise with hypertrophy of tapetal cells and pollen production in cold‐sensitive genotypes with insufficient levels of GA. Suppression of the cell wall‐bound acid invertase gene and the monosaccharide transporter gene disrupts sugar transport to the tapetum, leading to abnormal pollen development (Oliver et al. 2005; Mamun et al. 2006).

Both the cold‐tolerant and sensitive sorghum genotypes exhibited significant differences between optimal conditions and cold stress. Both responded equally sensitively to cold in BBCH39 with reduced GA concentrations. Maintaining the GA pool in BBCH35 and its decline in BBCH39, along with a reduced grain count (Neitzert et al. 2025), could suggest that maintaining a sufficiently high pool of GAs is less relevant for the ability to achieve full grain formation after reproductive cold stress, similar to the suppression of ABA production. Additionally, this supports the assumption that pollen fertility in sorghum is less restricted by cold and rather involves the sensitivity of the female reproductive organ (Osuna‐Ortega et al. 2003). In addition to the high pool of active gibberellins in both genotypes after cold stress in BBCH35, the high pollen fertility in the cold‐tolerant genotype after stress in sensitive stages (BBCH35 and BBCH39) despite a decline in GAs in BBCH39 contradicts the assumption that, as in rice, a reduction in GAs leads to abnormal pollen development. Furthermore, the correlation analysis showed that pollen traits were not correlated with yield parameters, supporting the conclusion that yield sensitivity to cold in sorghum is not driven by limitations in pollen development but rather by other, presumably female reproductive processes.

When comparing GA levels between the two genotypes, regardless of condition and stress, no differences were observed after exposure to cold. This suggests that the response of gibberellin synthesis to cold is very similar in both genotypes. Significant differences were observed in GA concentration between developmental stages. Here, the same trend is observed regardless of genotype and condition (stress and optimal conditions). In the sensitive stage BBCH35, GA concentration is highest in all cases, with a continuous decline in concentration towards BBCH51 (details in Section 4.4). This suggests that GA bottlenecks in sorghum are not the limiting factor for reproductive cold tolerance, and that breeding strategies aimed at stabilizing GA pools are likely less effective in sorghum than in rice.

Against this background, cold‐tolerant rice genotypes are known to maintain an adequate pool of bioactive gibberellins under cold stress, in part via altered biosynthetic pathways of GA4 and GA7 (Sharma and Nayyar 2016). In sorghum, however, the presence of GA4 and GA7 could not be confirmed in this experiment under either optimal conditions or after cold stress. Together with the similar GA profiles of the two genotypes examined, this supports the view that GA‐related bottlenecks are not the decisive factor for reproductive cold tolerance in sorghum.

### 4.3 Jasmonic Acid and Jasmonic Acid Isoleucine

Jasmonic acid (JA) and its biologically active form jasmonic acid isoleucine (JAIle) have been linked to cold stress in several studies. As a signaling molecule for stress responses, increased jasmonic acid production can prepare the plant for stress and is involved in the regulation of stress responses. In grasses, JA has been found to play a significant role in the development of generative tissue (Cai et al. 2014). The ICE‐CBF transcriptional regulation pathway has been identified as particularly important in maintaining plant development under cold stress conditions (Kim et al. 2015). In Arabidopsis, it has been demonstrated that the biosynthesis of the phytohormone regulates the expression of cold‐responsive genes through the CBF transcriptional pathway, which are responsible for improved cold tolerance (Hu et al. 2017). Further studies have shown that in rice, several genes related to JA biosynthesis (including *AOC*, *AOS1*, *AOS2*, and *LOX2*), as well as signaling genes (*COI1a* and *bHLH148*), respond positively to cold stress (Du et al. 2013; Hu et al. 2017). Furthermore, jasmonic acid plays a role in protecting against pests and diseases by inducing defense mechanisms, which may help protect the plant during the cold tolerance process (Chini et al. 2016; Zhang et al. 2017; Al‐Zahrani et al. 2020). An increased production of jasmonic acid by pests is excluded in this experiment due to controlled conditions in the climate chamber experiment.

In this study, no significant differences were observed in the concentration of JA and JAIle between the two genotypes under optimal conditions and cold stress across all developmental stages. Also, no differences were observed between the genotypes in the sensitive stages (BBCH35 and BBCH39), regardless of condition. Contrary to expectations, based on these results, it can be summarized that the accumulation of jasmonic acid in cold stress in sorghum seems to be low compared to other grasses (Cai et al. 2014; Du et al. 2013). With the absence of increased JA accumulation, the expression of JA biosynthesis genes at low temperatures in sorghum, unlike in rice (Du et al. 2013), does not seem to be induced by cold. To test this hypothesis, quantitative gene expression analyses (qPCR) or transcriptome analyses would be useful to determine whether JA biosynthesis genes are indeed unresponsive to cold in sorghum.

However, when comparing between developmental stages after cold stress, the cold‐sensitive genotype shows significantly lower JA concentrations in stages BBCH35 and BBCH39 compared to BBCH51. In contrast, the cold‐tolerant genotype maintains a consistent level of JA across all stages investigated. It can be assumed that there is generally a sufficient level of JA in sorghum to control the processes necessary for maintaining plant development under cold stress (Kim et al. 2015). Also, concerning the concentration of JAIle, significant differences between developmental stages exist. Here, regardless of genotype and condition (stress and optimal conditions), the same trend is observed. In the sensitive stages BBCH35 and BBCH39, the concentration of JAIle is lower in all cases than in BBCH51, with a continuous increase with the plant's age. The consistent trend of JAIle in the cold‐tolerant genotype and the absence of differences from optimal conditions to cold stress support the assumption that there seems to be a sufficiently high level of jasmonic acid and its derivatives in sorghum to ensure plant development under cold conditions. However, this does not provide an explanation for the lack of differences between cold‐tolerant and cold‐sensitive genotypes. In addition to JAIle, the derivative methyl jasmonate (MeJA) can also be formed in the cytoplasm from jasmonic acid. MeJA has already been associated with reduced cold damage in *Curcurbita pepo L*. (Wang and Buta 1994). In the future, investigating the MeJA derivative could provide insights into the role of JA and its derivatives.

### 4.4 JA‐GA Cross‐Talk

Jasmonic acid (JA), the defense signal, and gibberellins (GA), the hormone required for growth, interact in the form of antagonistic or synergistic influence in several signaling pathways. This allows the plant to adjust growth and adaptation to the prevailing environmental conditions. Several studies have shown that there is an interaction between JA and GA under both normal and stress conditions (Achard et al. 2008; Um et al. 2018).

In *Arabidopsis thaliana* , it has been shown that under normal conditions, accumulation of GA leads to the degradation of DELLA proteins. Under cold stress, the GA level is reduced, leading to an accumulation of DELLA proteins (Achard et al. 2008). DELLA proteins activate by binding to JAZ proteins, which regulate jasmonate‐responsive genes (Wingler et al. 2020).

Our results suggest the existence of an antagonistic interaction between JAIle, the biologically active form of JA, and GA concentration in Sorghum. While the JAIle concentration increases with the age of the plant regardless of whether the plant is exposed to optimal conditions or stress, the GA concentration decreases progressively, reaching its peak at the beginning of the early reproductive phase. This is evidence that antagonistic JA‐GA crosstalk occurs in Sorghum under both control and stress conditions. However, the extent to which this cross‐talk regulates plant growth and development under stress conditions needs to be further elucidated in additional experiments.

## 5 Outlook

The results of this study lay a foundation for elucidating the physiological mechanisms underlying reproductive cold tolerance in sorghum. The focus was on analyzing phytohormone dynamics under cold stress.

A limiting factor of this study is the number of biological replicates: for each treatment and developmental stage, three independent plants were available, each analyzed in two technical replicates to increase measurement precision. A higher number of biological replicates would further strengthen the statistical power. Nevertheless, the results provide robust insights into hormonal dynamics during the reproductive cold stress response and form a solid basis for further investigations.

To gain a more comprehensive understanding of the mechanisms underlying cold tolerance, additional approaches are necessary. Transcriptome analyses, for example, could provide insights into the molecular basis, while physiological measurements such as flag leaf photosynthetic activity or chlorophyll content could help link hormonal responses to physiological adaptations. The observed differences in ABA, GA, and JA dynamics provide valuable hypotheses for future studies in which hormone profiling, gene expression data, and physiological measurements are integrated.

Hormone profiles or the expression of hormone‐related genes may serve as indirect selection markers—particularly at early developmental stages—potentially accelerating selection in practical breeding programs.

## 6 Conclusion

A deeper understanding of reproductive cold tolerance is crucial for expanding sorghum cultivation into temperate regions. This study demonstrates that cold‐tolerant sorghum is capable of downregulating ABA concentration under cold stress. Contrary to the literature on rice, which often links elevated ABA levels and insufficient pools of bioactive gibberellins in sensitive genotypes to abnormal pollen development, this does not appear to be the case in sorghum. Additionally, an antagonistic interaction between GA and JA was observed, independent of genotype and environmental conditions. These findings contribute to a better understanding of the physiological mechanisms of cold tolerance in sorghum and may provide valuable insights for future breeding efforts aimed at accelerating the spread of cold‐tolerant sorghum varieties in temperate climates.

## Author Contributions

L.N. planned and supervised the climate chamber experiments and data collection, performed the data analysis, interpreted the results, and wrote the manuscript. N.K. contributed to data analysis. Y.A.T.M. and N.W. carried out the phytohormone analyses and contributed to manuscript editing. R.S. received the funding, contributed to the study design, and edited the manuscript. B.W. received the funding, interpreted the results, contributed to the study design, and edited the manuscript. S.W. designed the study and edited the manuscript. All authors contributed to the article and approved the submitted version.

## Funding

The authors have nothing to report.

## Conflicts of Interest

The authors declare no conflicts of interest.

## Peer Review

The peer review history for this article is available in the Supporting Information for this article.

## Supporting information


**Figure S1:** Heatmap illustrating Pearsons correlations among the analyzed traits for the cold‐tolerant genotype SB14011 under optimal conditions: Abscisic acid (ABA), Abscisic acid glucose ester (ABAGe), Dihydrophaseic acid (DHPA), Jasmonic acid (JA).
**Figure S2:** Heatmap illustrating Pearsons correlations among the analyzed traits for the cold‐sensitive genotype SC1056 under optimal conditions: Abscisic acid (ABA), Abscisic acid glucose ester (ABAGe), Dihydrophaseic acid (DHPA), Jasmonic acid (JA).

**Data S1:** Peer Review.

**Data S2:** Supplementary Information.

## Data Availability

The data supporting the findings of this study are provided in the Supplementary Material of this article.

## References

[pld370133-bib-0001] Achard, P. , F. Gong , S. Cheminant , M. Alioua , P. Hedden , and P. Genschik . 2008. “The Cold‐Inducible CBF1 Factor‐Dependent Signaling Pathway Modulates the Accumulation of the Growth‐Repressing DELLA Proteins via Its Effect on Gibberellin Metabolism.” Plant Cell 20, no. 8: 2117–2129. 10.1105/tpc.108.058941. 18757556 PMC2553604

[pld370133-bib-0002] Al‐Zahrani, W. , S. O. Bafeel , and M. El‐Zohri . 2020. “Jasmonates Mediate Plant Defense Responses to *Spodoptera exigua* Herbivory in Tomato and Maize Foliage.” Plant Signaling & Behavior 15, no. 5: 1746898. 10.1080/15592324.2020.1746898. 32290765 PMC7238883

[pld370133-bib-0003] Balsevich, J. J. , A. J. Cutler , N. Lamb , et al. 1994. “Response of Cultured Maize Cells to (+)‐abscisic Acid, (−)‐abscisic Acid, and Their Metabolites.” Plant Physiology 106, no. 1: 135–142. 10.1104/pp.106.1.135. 12232311 PMC159508

[pld370133-bib-0004] Berenji, J. , and J. Dahlberg . 2004. “Perspectives of Sorghum in Europe.” Journal of Agronomy and Crop Science 190, no. 5: 332–338. 10.1111/j.1439-037X.2004.00102.x.

[pld370133-bib-0005] Bindraban, P. S. , M. van der Velde , L. Ye , et al. 2012. “Assessing the Impact of Soil Degradation on Food Production.” Current Opinion in Environmental Sustainability 4, no. 5: 478–488. 10.1016/j.cosust.2012.09.015.

[pld370133-bib-0006] Bohnert, H. J. , D. E. Nelson , and R. G. Jensen . 1995. “Adaptations to Environmental Stresses.” Plant Cell 7, no. 7: 1099–1111. 10.1105/tpc.7.7.1099. 12242400 PMC160917

[pld370133-bib-0007] Cai, Q. , Z. Yuan , M. Chen , et al. 2014. “Jasmonic Acid Regulates Spikelet Development in Rice.” Nature Communications 5: 3476. 10.1038/ncomms4476. 24647160

[pld370133-bib-0008] Casper, B. R. E. N. D. A. B. 1990. “Timing of Embryo Abortion and the Effect of Ovule Thinning on Nutlet Mass in *Cryptantha flava* (Boraginaceae).” Annals of Botany 65, no. 5: 489–492. 10.1093/oxfordjournals.aob.a087960.

[pld370133-bib-0009] Chakrabarty, S. , N. Kravcov , A. Schaffasz , R. J. Snowdon , B. Wittkop , and S. Windpassinger . 2021. “Genetic Architecture of Novel Sources for Reproductive Cold Tolerance in *Sorghum* .” Frontiers in Plant Science 12: 772177. 10.3389/fpls.2021.772177. 34899798 PMC8652046

[pld370133-bib-0010] Chini, A. , S. Gimenez‐Ibanez , A. Goossens , and R. Solano . 2016. “Redundancy and Specificity in Jasmonate Signalling.” Current Opinion in Plant Biology 33: 147–156. 10.1016/j.pbi.2016.07.005. 27490895

[pld370133-bib-0011] Cutler, A. J. , and J. E. Krochko . 1999. “Formation and Breakdown of ABA.” Trends in Plant Science 4, no. 12: 472–478. 10.1016/S1360-1385(99)01497-1. 10562731

[pld370133-bib-0012] Downes, R. W. , and D. R. Marshall . 1971. “Low Temperature Induced Male Sterility in *Sorghum bicolor* .” Australian Journal of Experimental Agriculture 11, no. 50: 352. 10.1071/EA9710352.

[pld370133-bib-0013] Du, H. , H. Liu , and L. Xiong . 2013. “Endogenous Auxin and Jasmonic Acid Levels Are Differentially Modulated by Abiotic Stresses in Rice.” Frontiers in Plant Science 4: 397. 10.3389/fpls.2013.00397. 24130566 PMC3793129

[pld370133-bib-0014] Dumas, C. , R. B. Knox , and T. Gaude . 1984. “Pollen—pistil recognition: new concepts from electron microscopy and cytochemistry.” In International Review of Cytology, vol. 90, 239–272. Academic Press.

[pld370133-bib-0015] Garbero, M. , H. Pedranzani , F. Zirulnik , et al. 2011. “Short‐Term Cold Stress in two Cultivars of Digitaria Eriantha: Effects on Stress‐Related Hormones and Antioxidant Defense System.” Acta Physiologiae Plantarum 33, no. 2: 497–507. 10.1007/s11738-010-0573-z.

[pld370133-bib-0016] Gothandam, K. M. , E. S. Kim , and Y. Y. Chung . 2007. “Ultrastructural Study of Rice Tapetum Under Low‐Temperature Stress.” Journal Of Plant Biology 50, no. 4: 396–402. 10.1007/BF03030674.

[pld370133-bib-0017] Guo, Z. , D. Chen , and T. Schnurbusch . 2015. “Variance Components, Heritability and Correlation Analysis of Anther and Ovary Size During the Floral Development of Bread Wheat.” Journal of Experimental Botany 66, no. 11: 3099–3111. 10.1093/jxb/erv117. 25821074

[pld370133-bib-0018] Hu, Y. , Y. Jiang , X. Han , H. Wang , J. Pan , and D. Yu . 2017. “Jasmonate Regulates Leaf Senescence and Tolerance to Cold Stress: Crosstalk With Other Phytohormones.” Journal of Experimental Botany 68, no. 6: 1361–1369. 10.1093/jxb/erx004. 28201612

[pld370133-bib-0019] Huang, D. , W. Wu , S. R. Abrams , and A. J. Cutler . 2008. “The Relationship of Drought‐Related Gene Expression in Arabidopsis Thaliana to Hormonal and Environmental Factors.” Journal of Experimental Botany 59, no. 11: 2991–3007. 10.1093/jxb/ern155. 18552355 PMC2504347

[pld370133-bib-0020] Ji, X. , B. Dong , B. Shiran , et al. 2011. “Control of Abscisic Acid Catabolism and Abscisic Acid Homeostasis Is Important for Reproductive Stage Stress Tolerance in Cereals.” Plant Physiology 156, no. 2: 647–662. 10.1104/pp.111.176164. 21502188 PMC3177265

[pld370133-bib-0021] Kim, Y. S. , M. Lee , J.‐H. Lee , H.‐J. Lee , and C.‐M. Park . 2015. “The Unified ICE‐CBF Pathway Provides a Transcriptional Feedback Control of Freezing Tolerance During Cold Acclimation in *Arabidopsis* .” Plant Molecular Biology 89, no. 1–2: 187–201. 10.1007/s11103-015-0365-3. 26311645

[pld370133-bib-0022] Krochko, J. E. , G. D. Abrams , M. K. Loewen , S. R. Abrams , and A. J. Cutler . 1998. “(+)‐abscisic Acid 8′‐Hydroxylase Is a Cytochrome P450 Monooxygenase.” Plant Physiology 118, no. 3: 849–860. 10.1104/pp.118.3.849. 9808729 PMC34795

[pld370133-bib-0023] Kushiro, T. , M. Okamoto , K. Nakabayashi , et al. 2004. “The Arabidopsis Cytochrome P450 CYP707A Encodes ABA 8′‐Hydroxylases: Key Enzymes in ABA Catabolism.” EMBO Journal 23, no. 7: 1647–1656. 10.1038/sj.emboj.7600121. 15044947 PMC391058

[pld370133-bib-0024] Li, K.‐L. , X. Bai , Y. Li , et al. 2011. “GsGASA1 Mediated Root Growth Inhibition in Response to Chronic Cold Stress Is Marked by the Accumulation of DELLAs.” Journal of Plant Physiology 168, no. 18: 2153–2160. 10.1016/j.jplph.2011.07.006. 21855169

[pld370133-bib-0025] Mamun, E. A. , S. Alfred , L. C. Cantrill , R. L. Overall , and B. G. Sutton . 2006. “Effects of Chilling on Male Gametophyte Development in Rice.” Cell Biology International 30, no. 7: 583–591. 10.1016/j.cellbi.2006.03.004. 16730464

[pld370133-bib-0026] Nambara, E. , and A. Marion‐Poll . 2005. “Abscisic Acid Biosynthesis and Catabolism.” Annual Review of Plant Biology 56: 165–185. 10.1146/annurev.arplant.56.032604.144046. 15862093

[pld370133-bib-0027] Neitzert, L. , N. Kravcov , B. Wittkop , R. Snowdon , and S. Windpassinger . 2025. “Reproductive Cold Stress in Contrasting Sorghum Genotypes: Is Pollen Fertility Really the Crucial Trait?” Plant Direct 9: e70065. 10.1002/pld3.70065. 40330702 PMC12050216

[pld370133-bib-0029] Oliver, S. N. , E. S. Dennis , and R. Dolferus . 2007. “ABA Regulates Apoplastic Sugar Transport and Is a Potential Signal for Cold‐Induced Pollen Sterility in Rice.” Plant and Cell Physiology 48, no. 9: 1319–1330. 10.1093/pcp/pcm100. 17693452

[pld370133-bib-0028] Oliver, S. N. , J. T. Van Dongen , S. C. Alfred , et al. 2005. “Cold‐induced repression of the rice anther‐specific cell wall invertase gene OSINV4 is correlated with sucrose accumulation and pollen sterility.” Plant, Cell & Environment 28, no. 12: 1534–1551.

[pld370133-bib-0030] Osuna‐Ortega, J. , M. D. C. Mendoza‐Castillo , and L. Mendoza‐Onofre . 2003. “Sorghum cold tolerance, pollen production.” Maydica 48: 125–132.

[pld370133-bib-0031] Patil, J. V. 2017. Millets and Sorghum. Wiley.

[pld370133-bib-0032] Ren, H. , Z. Gao , L. Chen , et al. 2007. “Dynamic Analysis of ABA Accumulation in Relation to the Rate of ABA Catabolism in Maize Tissues Under Water Deficit.” Journal of Experimental Botany 58, no. 2: 211–219. 10.1093/jxb/erl117. 16982652

[pld370133-bib-0033] Sakata, T. , S. Oda , Y. Tsunaga , et al. 2014. “Reduction of Gibberellin by low Temperature Disrupts Pollen Development in Rice.” Plant Physiology 164, no. 4: 2011–2019. 10.1104/pp.113.234401. 24569847 PMC3982758

[pld370133-bib-0034] Schaffasz, A. , S. Windpassinger , R. Snowdon , and B. Wittkop . 2019. “Reproductive Cold Stress Tolerance in Sorghum F1 Hybrids Is a Heterotic Trait.” Agronomy 9, no. 9: 508. 10.3390/agronomy9090508.

[pld370133-bib-0035] Setha, S. , S. Kondo , N. Hirai , and H. Ohigashi . 2005. “Quantification of ABA and Its Metabolites in Sweet Cherries Usingdeuterium‐Labeled Internal Standards.” Plant Growth Regulation 45, no. 3: 183–188. 10.1007/s10725-005-3088-7.

[pld370133-bib-0036] Sharma, K. D. , and H. Nayyar . 2016. “Regulatory Networks in Pollen Development Under Cold Stress.” Frontiers in Plant Science 7: 402. 10.3389/fpls.2016.00402. 27066044 PMC4814731

[pld370133-bib-0049] Šimura, J. , I. Antoniadi , J. Široká , et al. 2018. “Plant Hormonomics: Multiple Phytohormone Profiling by Targeted Metabolomics.” Plant Physiology 177, no. 2: 476–489. 10.1104/pp.18.00293. 29703867 PMC6001343

[pld370133-bib-0037] Srinivasan, A. , N. P. Saxena , and C. Johansen . 1999. “Cold Tolerance During Early Reproductive Growth of Chickpea (*Cicer Arietinum* L.): Genetic Variation in Gamete Development and Function.” Field Crops Research 60, no. 3: 209–222. 10.1016/S0378-4290(98)00126-9.

[pld370133-bib-0038] Stephens, J. C. , F. R. Miller , and D. T. Rosenow . 1967. “Conversion of Alien Sorghums to Early Combine Genotypes 1.” Crop Science 7, no. 4: 396. 10.2135/cropsci1967.0011183X000700040036x.

[pld370133-bib-0039] Um, T. Y. , H. Y. Lee , S. Lee , et al. 2018. “Jasmonate Zim‐Domain Protein 9 Interacts With Slender Rice 1 to Mediate the Antagonistic Interaction Between Jasmonic and Gibberellic Acid Signals in Rice.” Frontiers in Plant Science 9: 1866. 10.3389/fpls.2018.01866. 30619427 PMC6305323

[pld370133-bib-0040] Wang, C. Y. , and J. G. Buta . 1994. “Methyl Jasmonate Reduces Chilling Injury in Cucurbita pepo Through Its Regulation of Abscisic Acid and Polyamine Levels.” Environmental and Experimental Botany 34, no. 4: 427–432. 10.1016/0098-8472(94)90025-6.

[pld370133-bib-0041] Wang, M. , S. Hoekstra , S. van Bergen , et al. 1999. “Apoptosis in Developing Anthers and the Role of ABA in This Process During Androgenesis in *Hordeum vulgare* L.” Plant Molecular Biology 39, no. 3: 489–501. 10.1023/A:1006198431596. 10092177

[pld370133-bib-0042] Wingler, A. , V. Tijero , M. Müller , B. Yuan , and S. Munné‐Bosch . 2020. “Interactions Between Sucrose and Jasmonate Signalling in the Response to Cold Stress.” BMC Plant Biology 20, no. 1: 176. 10.1186/s12870-020-02376-6. 32321430 PMC7178619

[pld370133-bib-0043] Wood, A. W. , D. K. Y. Tan , E. A. Mamun , and B. G. Sutton . 2006. “Sorghum can Compensate for Chilling‐Induced Grain Loss.” Journal of Agronomy and Crop Science 192, no. 6: 445–451. 10.1111/j.1439-037X.2006.00233.x.

[pld370133-bib-0044] Zadoks, J. C. , T. T. Chag , and C. F. Konzak . 1974. “A Decimal Code for the Growth Stages of Cereals.” Weed Research 14: 415–421.

[pld370133-bib-0045] Zeevaart, J. A. 1999. “Abscisic acid metabolism and its regulation.” In New Comprehensive Biochemistry, vol. 33, 189–207. Elsevier.

[pld370133-bib-0046] Zhang, L. , F. Zhang , M. Melotto , J. Yao , and S. Y. He . 2017. “Jasmonate Signaling and Manipulation by Pathogens and Insects.” Journal of Experimental Botany 68, no. 6: 1371–1385. 10.1093/jxb/erw478. 28069779 PMC6075518

[pld370133-bib-0047] Zheng, L.‐Y. , X.‐S. Guo , B. He , et al. 2011. “Genome‐Wide Patterns of Genetic Variation in Sweet and Grain sorghum (*Sorghum Bicolor*).” Genome Biology 12, no. 11: R114. 10.1186/gb-2011-12-11-r114. 22104744 PMC3334600

