# Supplementary material for: Cold Shock for Cold Tolerance: Phytohormone Dynamics in Sorghum Provides Insights
Source: Plant Direct. 2026 Jan 15;10(1):e70133. doi: 10.1002/pld3.70133 (PMC12807585; doi:10.1002/pld3.70133)
Supplement: Supplementary file 2 — Data S1: Peer Review. [file PLD3-10-e70133-s001.pdf]

Cold Shock for Cold Tolerance: Phytohormone Dynamics in Sorghum Provides Insights

Luisa Neitzert, Natalja Kravcov, Steffen Windpassinger , Rod J Snowdon, Benjamin Wittkop, Nicolaus von Wirén , Yudelsy Antonia Tandron Moya.

Decision Letter Round 1:

August 27, 2025

Luisa Neitzert

Department of Plant breeding

Gießen

Germany

RE: Cold Shock for Cold Tolerance: Phytohormone Dynamics in Sorghum Provides Insights

Dear Dr. Neitzert:

Thank you for submitting to Plant Direct. All required reviews have been returned and we have now finished our evaluation of your manuscript. In light of the reviewers' and editor's comments, further revisions are needed before the paper can be accepted for publication in Plant Direct.

Please view the editors' and reviewers' comments below and use their suggestions as a guide while you work on your revision.

When uploading the revised version of this article, please be sure to include the following:

-A word document that contains your response to the reviewers. You should respond to each reviewer comment and note the changes made to the manuscript. If you do not agree with a reviewer's comment and choose not to make a suggested revision, please explain why. Please try to provide as complete an answer as possible to each reviewer's criticisms in the "response to reviewers" section.

-A tracked changes document

- A clean version of the latest version of the manuscript

Thank you for your submission. The reviewers highlight many strengths of the manuscript. I recommend that you carefully revisit the points highlighted by the

reviewers to adjust the interpretation of the results to align with the experiments completed. Reviewer #1 highlights strategies for this for most points.

To upload your revision, please click the link below.

https://plantdirect.msubmit.net/cgi-bin/main.plex?el=A7Lr2oq5A2nEB7I5A9ftdJjNpO8kzUmHWdAgqsELsHgZ

In order to provide as timely a service as possible, we ask that your revision is resubmitted within three months after receipt of this request. If an extension is needed, please send a request, along with a brief explanation, to the editorial office at plantdirect@wiley.com .

Thank you very much for giving us an opportunity to review your work. I look forward to receiving the next version.

Sincerely,

Colleen Doherty

-- Editor comments:

Thank you for your submission. The reviewers highlight many strengths of the manuscript. I recommend that you carefully revisit the points highlighted by the reviewers to adjust the interpretation of the results to align with the experiments completed. Reviewer #1 highlights strategies for this for most points.

-- Reviewer comments:

Reviewer #2:

This manuscript addresses an important and timely question: the physiological and hormonal mechanisms underlying reproductive cold tolerance in sorghum. The topic is highly relevant given the need to expand sorghum cultivation into temperate regions under changing climates. The experimental design-comparing cold-tolerant and cold-sensitive inbred lines at defined developmental stages under controlled cold stress-is sound, and the application of UHPLC-HESI-HRMS for hormone profiling provides robust quantitative data.

The study generates novel insights, particularly regarding ABA downregulation in tolerant genotypes, differences in GA and JA regulation, and the potential involvement of female fertility in cold sensitivity. However, some aspects of the manuscript require clarification and strengthening before publication. The main concerns relate to limited replication, mechanistic overinterpretation without molecular validation, and the need for a more focused and concise discussion.

Overall, the study is suitable for publication after major revision.

Major Points

Replication and Statistical Power

The use of only three biological replicates per treatment and stage is a concern. While technical replicates add precision, biological replication is critical for hormone analyses. The authors should acknowledge this limitation explicitly and discuss how it may affect interpretation.

Mechanistic Interpretation Without Gene Expression Data

Many conclusions about hormone regulation (e.g., ABA hydroxylation, GA biosynthesis, JA pathway regulation) are speculative without supporting gene expression data. Unless transcriptomic or qPCR data can be added, the discussion should be carefully rephrased to avoid overinterpretation.

Reproductive Sensitivity: Pollen vs. Female Fertility

The manuscript argues that female fertility impairment may drive cold sensitivity, but direct evidence is lacking. If no data are available, this hypothesis should be presented more cautiously as a possible explanation rather than a firm conclusion.

Field Relevance

All experiments were conducted under controlled climate chamber conditions. While this ensures reproducibility, field-level validation is essential for practical breeding relevance. The authors should note this limitation and recommend follow-up studies.

Discussion Streamlining

The discussion is informative but at times repetitive, especially regarding ABA pathways in rice vs. sorghum. A more concise presentation highlighting the novel contributions of this study (e.g., sorghum-specific differences, implications for breeding) would strengthen the impact.

Minor Points

Figures and Tables

Some figures (e.g., hormone profiles with many p-values) are dense. Simplifying or summarizing key findings would improve clarity.

Ensure all figure legends are fully self-explanatory.

Terminology

Consistently use "ABA glucose ester (ABAGe)" throughout (sometimes written as ABAGle in the manuscript).

Clarify abbreviations at first use in each section.

References

The discussion cites several rice and Arabidopsis studies; ensure that the most recent and relevant references are included.

Double-check consistency in reference formatting.

Language and Style

The manuscript is generally well written, but some sentences in the discussion are long and could be broken down for clarity. Minor editing for conciseness is recommended.

Reviewer #3:

This research addresses an important problem in sorghum production: the limitation of cultivation in temperate regions due to cold stress, particularly during the reproductive phase. Focusing on different reproductive stages and phytohormonal regulation (ABA, gibberellins, and jasmonic acid) is relevant and potentially useful for breeding programs. However, the manuscript requires significant grammatical and English editing improvements to improve clarity and readability. Additionally, the results and discussion are currently too descriptive without clearly highlighting the novelty, broader

impact, or integration with plant physiology. The following comments provide specific points for improvement.

Major Concerns

The manuscript contains multiple grammatical issues and phrasing issues. Sentences are long and sometimes redundant.

Ex: Line 48. (Nambara und Marion-Poll 2005) and (Sharma und Nayyar 2016) should be as (Nambara and Marion-Poll 2005) and (Sharma and Nayyar 2016). It is was repeated at several places in the manuscript.

Repetition of phrases like "cold-tolerant" and "cold-sensitive" genotypes could be reduced for conciseness.

Line 43-44: Gene names should be italicized.

Methods:

Line 84: Describe the acronym for these (BBCH35, 84 BBCH39, and BBCH51) and cite the proper reference. And BBCH stands for?

The methods section, growth chamber experiments section, and phytohormone analysis section need to cite suitable references for the obtained methods.

The methods section must mention details of chemical standards used for each hormone and its derivatives. The liquid phase is also used for specific hormone analysis in the UHPLC-HESI-HRMS.

Provide the full forms for any acronyms or short forms used in the manuscript (Ex. UHPLC-HESI-HRMS).

Line 156-157: Not clear. Where is the information for other hormone compounds?

The same experiment results were performed with a different analysis. What is the basis for this, and what is its significance?

Results:

The results describe the hormones measured and the observations in detail, but this level of descriptive reporting is not necessary; the results section can be condensed and highlights the trends between the genotypes and reproductive stages. No need to write individually write for each hormone in detail if not significant.

The manuscript should emphasize the research question, novelty, and significance rather than catalog detailed findings. For example, the antagonistic interaction between

gibberellins and jasmonic acid could be condensed into a single impactful statement highlighting its potential relevance. Similar things works for other hormone parts as well.

While phytohormone dynamics under stress are important, the manuscript does not clarify whether these hormonal regulations are correlated with any physiological changes in the reproductive organs.

The authors should articulate how this study provides new insights specific to sorghum reproductive cold tolerance that were not already known from other systems. For example, "contrary to established reports in cereals, we found..." would strengthen the novelty claim.

Hormone profiling alone is insufficient to explain cold tolerance mechanisms. The study would be stronger if the hormonal dynamics were connected to physiological responses or molecular gene expression data, such as: Flag leaf photosynthetic activity or chlorophyll content under stress, pollen fertility, or grain set/yield data under cold shock.

The study would benefit from including hormone biosynthesis, hormone-regulated genes responsible for reproductive organ development, or agronomic trait-related gene expression data. This would provide a mechanistic basis for the hormonal changes observed rather than relying solely on metabolite levels.

Clearly state the novel contribution in the discussion: what is different about sorghum than other crops, and why these matters for breeding cold tolerance.

Decision Letter Round 2:

December 5, 2025

Luisa Neitzert

Department of Plant breeding

Gießen

Germany

MSID: 2025-01803R1

MS TITLE: Cold Shock for Cold Tolerance: Phytohormone Dynamics in Sorghum Provides Insights

Dear Dr. Luisa Neitzert:

I am pleased to inform you that your manuscript "Cold Shock for Cold Tolerance: Phytohormone Dynamics in Sorghum Provides Insights" has been accepted for publication in Plant Direct.

Your article will appear online in the next available issue of Plant Direct. To ensure your article gets published as quickly as possible, please pay attention to the steps detailed below. We have found that most of the delays happen at this stage, especially at the payment stage, so please respond as quickly as possible when prompted.

License Agreement: Once your article has been accepted it will move to Production and undergo admin and file checking - you may receive an email with any queries we have at this stage. When all required items are received by the publisher and queries resolved, the corresponding author will receive an email from Wiley's Author Services system which will ask them to log on at https://authorservices.wiley.com/bauthor and will present them with the appropriate license for completion. Your article cannot be published until both the signed license agreement and payment of the article fee have been received.

Payment of the Open Access Article Publication Fee: All articles published in Plant Direct are fully open access: immediately and freely available to read, download and share. Plant Direct charges a publication fee to cover the publication costs. The corresponding author for this manuscript should have already received a quote from the payments team (cs-openaccess@wiley.com) with the estimated article publication fee; please email cs-openaccess@wiley.com if this has not been received. The corresponding author should log on to the Wiley Author Services site, where the publication fee can be paid by credit card or an invoice. Pro Forma can also be requested. Payment of the publication charge must be received before the article will be published online.

Proofs: You will have the opportunity to look over your paper once more when you receive the author proofs for your article. The proofs will be with you in approximately two weeks. Please note that, in addition to publishing reviewer comments, the author's responses to review comments will also be published alongside the final version of the paper. If you would not like the author's responses to be published, please contact the editorial office at plantdirect@wiley.com .

Promotion of your article: You can help your research get the attention it deserves! Check out Wiley's free Promotion Guide for best-practice recommendations for promoting your work at www.wileyauthors.com/eeo/guide. And learn more about Wiley

Editing Services which offers professional video, design, and writing services to create shareable video abstracts, infographics, conference posters, lay summaries, and research news stories for your research at www.wileyauthors.com/eeo/promotion.

Thank you again for your contribution to Plant Direct. If you have any questions, feel free to contact the editorial office at plantdirect@wiley.com .

Sincerely,

Colleen Doherty

Esther van der Knaap

Editor, Plant Direct

-- Editor comments

-- Reviewer comments:

Reviewer #2:

The authors satisfied all the comments raised by me.

Response Letter Round 1:

Response to the Reviewer

Reviewer #2:

This manuscript addresses an important and timely question: the physiological and hormonal

mechanisms underlying reproductive cold tolerance in sorghum. The topic is highly relevant given the

need to expand sorghum cultivation into temperate regions under changing climates. The

experimental design-comparing cold-tolerant and cold-sensitive inbred lines at defined

developmental stages under controlled cold stress-is sound, and the application of UHPLC-HESIHRMS for hormone profiling provides robust quantitative data.

The study generates novel insights, particularly regarding ABA downregulation in tolerant genotypes,

differences in GA and JA regulation, and the potential involvement of female fertility in cold

sensitivity. However, some aspects of the manuscript require clarification and strengthening before

publication. The main concerns relate to limited replication, mechanistic overinterpretation without

molecular validation, and the need for a more focused and concise discussion.

Overall, the study is suitable for publication after major revision.

Major Points

Replication and Statistical Power

The use of only three biological replicates per treatment and stage is a concern. While technical

replicates add precision, biological replication is critical for hormone analyses. The authors should

acknowledge this limitation explicitly and discuss how it may affect interpretation.

Answer: Thank you for this important comment. We agree that the number of biological replicates is

a limiting factor for the interpretability of hormone analyses. In our experimental design, we included

three biological replicates (three independent plants) per treatment and developmental stage. To

further increase precision, we analyzed two technical replicates per plant. We have explicitly

highlighted the limited number of biological replicates in the discussion and addressed the potential

limitations this imposes on the interpretation of our results.

Mechanistic Interpretation Without Gene Expression Data

Many conclusions about hormone regulation (e.g., ABA hydroxylation, GA biosynthesis, JA pathway

regulation) are speculative without supporting gene expression data. Unless transcriptomic or qPCR

data can be added, the discussion should be carefully rephrased to avoid overinterpretation.

Answer: We agree that, without complementary gene expression data, mechanistic interpretations of

hormone regulation (e.g., ABA hydroxylation, GA biosynthesis, JA regulation) remain limited.

Therefore, we have revised the corresponding passages in the discussion and, where not already

done, have phrased them more cautiously. In a separate section (Outlook), we have also explicitly

highlighted this point again.

Reproductive Sensitivity: Pollen vs. Female Fertility

The manuscript argues that female fertility impairment may drive cold sensitivity, but direct evidence

is lacking. If no data are available, this hypothesis should be presented more cautiously as a possible

explanation rather than a firm conclusion.

Answer: We have formulated the hypothesis in a more cautious manner.

Field Relevance

All experiments were conducted under controlled climate chamber conditions. While this ensures

reproducibility, field-level validation is essential for practical breeding relevance. The authors should

note this limitation and recommend follow-up studies.

Answer: We understand your point that field trials are important for assessing the practical breeding

relevance. However, the focus of the present study was on understanding the fundamental hormonal

mechanisms underlying cold tolerance. Field experiments on this topic are challenging, as plants do

not experience cold stress uniformly at the same developmental stages under natural conditions, and

additional stressors (e.g., pest infestation) could influence phytohormone regulation. For this reason,

we deliberately conducted the experiments in climate chambers to ensure comparable conditions..

Discussion Streamlining

The discussion is informative but at times repetitive, especially regarding ABA pathways in rice vs.

sorghum. A more concise presentation highlighting the novel contributions of this study (e.g.,

sorghum-specific differences, implications for breeding) would strengthen the impact.

Answer: Thank you for the comment. We would like to maintain the length of the discussion, as the

information is important for a proper interpretation of our results. Comparisons with rice are

particularly relevant, as there are currently very few data on reproductive cold tolerance in sorghum.

Since rice and sorghum are closely related, we consider it useful to present the current state of

research on rice in detail and to compare our results with it. In a new section, “Outlook,” we also

specifically address the breeding relevance.

Minor Points

Figures and Tables

Some figures (e.g., hormone profiles with many p-values) are dense. Simplifying or summarizing key

findings would improve clarity.

Ensure all figure legends are fully self-explanatory.

Answer: Thank you for the comment, with which we agree. We have condensed the results section to

improve clarity. However, we have retained the detailed representations, as we consider the p-values

relevant for the interpretation of the results.

Terminology

Consistently use "ABA glucose ester (ABAGe)" throughout (sometimes written as ABAGle in the

manuscript).

Clarify abbreviations at first use in each section.

Answer: We have standardized the terminology throughout the manuscript and consistently use

“ABA-glucose ester (ABAGe).” In addition, all abbreviations are now defined at their first occurrence

in each section.

References

The discussion cites several rice and Arabidopsis studies; ensure that the most recent and relevant

references are included.

Double-check consistency in reference formatting.

Answer: Thank you for the comment. We are aware that some of the cited references are older. This

is due to the fact that reproductive cold tolerance and cold sensitivity in sorghum is a very specific

topic that has so far been only scarcely studied. The available literature is also limited for other plant

species. We have therefore carefully searched for and integrated the best available references in

order to support our results as robustly as possible.

Language and Style

The manuscript is generally well written, but some sentences in the discussion are long and could be

broken down for clarity. Minor editing for conciseness is recommended.

Reviewer #3:

This research addresses an important problem in sorghum production: the limitation of cultivation in

temperate regions due to cold stress, particularly during the reproductive phase. Focusing on

different reproductive stages and phytohormonal regulation (ABA, gibberellins, and jasmonic acid) is

relevant and potentially useful for breeding programs. However, the manuscript requires significant

grammatical and English editing improvements to improve clarity and readability. Additionally, the

results and discussion are currently too descriptive without clearly highlighting the novelty, broader

impact, or integration with plant physiology. The following comments provide specific points for

improvement.

Major Concerns

The manuscript contains multiple grammatical issues and phrasing issues. Sentences are long and

sometimes redundant. Ex: Line 48. (Nambara und Marion-Poll 2005) and (Sharma und Nayyar 2016)

should be as (Nambara and Marion-Poll 2005) and (Sharma and Nayyar 2016). It is was repeated at

several places in the manuscript.

Repetition of phrases like "cold-tolerant" and "cold-sensitive" genotypes could be reduced for

conciseness.

Answer: Thank you for the comment. We have comprehensively revised the manuscript:

Sentence length & redundancies: Overly long and/or nested sentences were split, redundant

formulations removed, and unnecessary repetitions deleted.

Consistent citation style: All in-text citations were standardized to the English “and” (e.g., “Nambara

and Marion-Poll 2005,” “Sharma and Nayyar 2016”). In places where “und” had inadvertently been

used (e.g., formerly in line 48), this was corrected.

Reduction of repetitions (“cold-tolerant”/“cold-sensitive”): We have tried to repeat as little as

possible. In some cases, however, it is relevant to mention or repeat the attribute again at the

beginning of a chapter so that the reader immediately knows what it is about.

Terminology & consistency: Hyphenation and capitalization (e.g., “cold-tolerant,” “cold-sensitive”)

were standardized.

All adjustments are traceable in the attached tracked-changes version. We are happy to make further

linguistic refinements if needed.

Line 43-44: Gene names should be italicized.

Answer: We have modified this accordingly.

Methods:

Line 84: Describe the acronym for these (BBCH35, 84 BBCH39, and BBCH51) and cite the proper

reference. And BBCH stands for?

Answer: Thank you for your comment. We have now provided a more detailed description in the text.

The BBCH scale is used for the standardized coding of phenological development stages in

monocotyledonous and dicotyledonous plants. It is based on the cereal scale developed by Zadoks et

al. and divides plant development into macro- and microstages. The extended BBCH scale commonly

used today was developed jointly by the Federal Biological Research Centre for Agriculture and

Forestry (BBA), the Federal Plant Variety Office (BSA), and the German Agricultural Industry

Association (IVA). The abbreviation BBCH is derived from these institutions: Bundesanstalt für Landund Forstwirtschaft, Bundessortenamt, and Chemische Industrie. The specific meanings of BBCH35,

BBCH39, and BBCH51 are already explained in the text.

The methods section, growth chamber experiments section, and phytohormone analysis section

need to cite suitable references for the obtained methods.

Answer: Thank you for this helpful comment, we fully agree. In the climate chamber experiments, we

followed the protocol described by Schaffasz et al. (2019), who conducted comparable studies on

reproductive cold stress in sorghum. We have included the corresponding reference in the Methods

section.

We have included the reference (Šimura, et al., 2018) in the “Reference List” section. It refers to the

method used for the extraction of the phytohormones.

For the analysis of the phytohormones, excluding GA, the described liquid chromatography-high

resolution mass spectrometry method is published for the first time in this manuscript. For this

reason, we have included detailed information for both chromatography and ionization conditions.

The method validation data are available but are not included, as they are out the scope of this

publication.

The liquid chromatography-mass spectrometry method used for the analysis of GAs has been

previously published in Myliaev, et al., 2022. This reference has been included in the “Reference List”

section.

The methods section must mention details of chemical standards used for each hormone and its

derivatives. The liquid phase is also used for specific hormone analysis in the UHPLC-HESI-HRMS.

Answer: Thank you for your comment. The details of chemical standards as well as solvents used in

the liquid phase have been included.

Provide the full forms for any acronyms or short forms used in the manuscript (Ex. UHPLC-HESIHRMS).

Answer: Thank you for your comment. We have corrected it accordingly.

Line 156-157: Not clear. Where is the information for other hormone compounds?

Answer: Thank you for the comment. We are not completely sure what is meant and will revise the

section accordingly; if anything remains unclear after the revision, please let us know.

The same experiment results were performed with a different analysis. What is the basis for this,

and what is its significance?

Answer: Thank you for this comment. The different statistical tests were deliberately chosen, as each

addresses distinct biological questions. The Welch two-sample t-test was used to examine the effects

of cold stress compared to optimal conditions within the same genotype and developmental stage.

The paired t-test was applied to assess differences between the two genotypes within the same stage

and condition. In contrast, the one-way ANOVA allowed us to analyze differences between

developmental stages (BBCH35, BBCH39, BBCH51) within a given genotype and condition.

The combination of these approaches enables us to consider the effects of stress, genotype, and

developmental stage in a differentiated manner, thereby providing a more comprehensive picture of

hormonal regulation under cold stress.

Results:

The results describe the hormones measured and the observations in detail, but this level of

descriptive reporting is not necessary; the results section can be condensed and highlights the trends

between the genotypes and reproductive stages. No need to write individually write for each

hormone in detail if not significant.

The manuscript should emphasize the research question, novelty, and significance rather than

catalog detailed findings. For example, the antagonistic interaction between gibberellins and

jasmonic acid could be condensed into a single impactful statement highlighting its potential

relevance. Similar things works for other hormone parts as well.

Answer: Thank you for the comment, with which we agree. We have streamlined the results section

to focus on the essential information. In contrast, we have kept the discussion more detailed, as we

consider the information provided there important for understanding the conclusions.

While phytohormone dynamics under stress are important, the manuscript does not clarify whether

these hormonal regulations are correlated with any physiological changes in the reproductive organs.

The authors should articulate how this study provides new insights specific to sorghum reproductive

cold tolerance that were not already known from other systems. For example, "contrary to

established reports in cereals, we found..." would strengthen the novelty claim.

Answer: Thank you for the helpful comment. We have revised the manuscript accordingly and made

the correlation between hormone dynamics and reproductive physiology more explicit, and we have

articulated the novelty of our findings relative to rice/other cereals more clearly. In addition, we have

now included a new correlation analysis linking phytohormone concentrations with pollen and yield

parameters from a previously published study (Section 3.4) and integrated the results into the

discussion.

Hormone profiling alone is insufficient to explain cold tolerance mechanisms. The study would be

stronger if the hormonal dynamics were connected to physiological responses or molecular gene

expression data, such as: Flag leaf photosynthetic activity or chlorophyll content under stress, pollen

fertility, or grain set/yield data under cold shock.

The study would benefit from including hormone biosynthesis, hormone-regulated genes responsible

for reproductive organ development, or agronomic trait-related gene expression data. This would

provide a mechanistic basis for the hormonal changes observed rather than relying solely on

metabolite levels.

Answer: Thank you for the comment. We acknowledge that linking hormone dynamics with

physiological parameters or molecular gene expression data would further strengthen the study.

However, due to limited resources, such extensive follow-up experiments could not be conducted

within the scope of the present work. We have nevertheless pointed out in the discussion that such

complementary analyses would be valuable and necessary in future studies to achieve a more

comprehensive understanding of the mechanisms of cold tolerance.

Clearly state the novel contribution in the discussion: what is different about sorghum than other

crops, and why these matters for breeding cold tolerance.

Answer: Thank you for the comment. We have taken this into account during the revision
